# Physically Consistent Risk Calibration for Open-World Alarm Filtering in Distributed Optical Fiber Sensing

**DOI:** 10.3390/s26165174

**Published:** 2026-08-15

**Authors:** Qingmin Hou, Hanyang Zhang, Guanghua Xiao, Peng Zhang, Ziguang Jia

**Affiliations:** 1 School of Smart City, Chengdu Vocational & Technical College of Industry, Chengdu 610213, China; sspawn@163.com (Q.H.); xiaoguanghua_hit@163.com (G.X.); 2 College of Computer Science, Sichuan University, Chengdu 610065, China; 2024323042001@stu.scu.edu.cn; 3 Department of Civil Engineering, Dalian Maritime University, Dalian 116026, China; peng.zhang47@dlmu.edu.cn; 4 School of Chemical Engineering, Ocean and Life Sciences, Dalian University of Technology, Panjin 124221, China

**Keywords:** distributed optical fiber sensing, ϕ-OTDR, distributed acoustic sensing, open-world recognition, conformal prediction, false-alarm rate, physics-informed measurement, intrusion detection, sensor reliability

## Abstract

Distributed optical fiber sensing (DOFS) based on phase-sensitive optical time-domain reflectometry (ϕ-OTDR) is increasingly used for perimeter and pipeline monitoring, yet most recognizers are evaluated as closed-set classifiers, whereas a deployed fiber also records nuisance sources and event types absent from training. In our experiments, a closed-set recognizer with 0.999 accuracy still gives a false-alarm rate (FAR) of 0.42–0.64 on simulated unknown nuisance classes, and a conformal threshold calibrated only on known negatives does not remove this failure. We therefore propose Physically Consistent Risk Calibration (PCRC), which combines a label-free physical-consistency gate computed from spatial compactness, common-mode ratio, and signal-to-noise ratio (SNR) with a conformal threshold for gate-passing negatives and a three-level alarm/review/discard decision. The guarantee is conditional: conformal calibration controls gate-passing negatives under exchangeability, and the gate removes only physically inadmissible nuisance windows. On three public distributed acoustic sensing (DAS) field datasets, the known-negative FAR follows the target level when calibration and test negatives are exchangeable, held-out threat coverage reaches 0.87 and 0.68 on the two multichannel corpora, and every physically admissible held-out nuisance passes the gate and requires recalibration from verified field negatives. In a controlled simulation whose unknown nuisance classes are constructed to be inadmissible, PCRC produces no observed automatic false alarms while retaining 0.998 known-threat recall.

## 1. Introduction

Distributed optical fiber sensing (DOFS) transforms an ordinary telecom fiber into a dense array of vibration/strain transducers. Phase-sensitive optical time-domain reflectometry (ϕ-OTDR) and the broader family of distributed acoustic sensing (DAS) provide continuous, electrically passive monitoring over tens of kilometers and have become a standard instrument for perimeter security, pipeline integrity, and railway and border surveillance [1,2]. A raw acquisition is naturally a *space-time* matrix $x\in R^{N_{z}\times N_{t}}$ indexing $N_{z}$ fiber channels and $N_{t}$ temporal samples, in which intrusion events appear as localized, propagating energy patterns.

Most event-recognition pipelines treat the task as *closed-set* classification: a deep network or a hand-crafted feature pipeline maps each window to one of *K* predefined categories (e.g., walking, digging, vehicle) [3,4,5]. Reported accuracies often exceed 95% on curated test splits. In operation, however, the same fiber records event types that are not represented during training. These include previously unseen threat mechanisms (e.g., mechanical drilling, fence climbing) and nuisance sources, such as animals, benign mechanical activity near the cable, common-mode electrical/optical interference, and laser-phase noise bursts. A closed-set classifier has no explicit option for these cases. It must assign them to one of the known classes, which can lead to missed intrusions or avoidable false alarms.

We treat this as a measurement-reliability problem rather than only a classification problem: an alarm decision should be reported with a specified and empirically checkable error rate. Two gaps motivate our work.

**Gap 1: Confidence ≠ reliability in the open world.** A classifier can be well calibrated on the distribution used for validation and still be overconfident on novel inputs. In our work a classifier with an in-distribution expected calibration error (ECE) of 0.024 assigns a mean softmax confidence of 0.845 to unknown inputs. Thresholding this confidence, or calibrating it by conformal prediction on known negatives only, does not by itself control the FAR on novel negative classes.

**Gap 2: Useful physical structure is often left outside the decision rule.** The space-time signature of a genuine near-fiber disturbance is constrained by wave propagation and coupling to the cable. It should be spatially localized, it should not appear as a nearly identical common-mode trace on all channels, and it should be separated from the local noise floor. These simple checks do not replace a recognizer, but they can reject artifacts that are difficult to handle by class posterior scores alone.

**Contributions.** We study DOFS monitoring as open-world alarm filtering and make the following contributions:

1. We define the known/unknown × threat/non-threat strata used in open-world alarm filtering and show how a conformal false-alarm-rate (FAR) guarantee calibrated on known negatives can fail on novel negative classes.
2. We introduce a consistency score P(x)∈[0,1] from three interpretable descriptors: spatial compactness, common-mode ratio, and signal-to-noise ratio. The score is used as a physics gate before conformal risk calibration and a three-tier Alarm/Review/Discard decision.
3. We give a finite-sample conformal FAR bound for gate-passing negatives and an FAR decomposition showing that windows rejected by the physics gate cannot produce automatic alarms. The resulting bound is conditional: it applies to novel nuisance sources only to the extent that they fall in the physically inadmissible region, an assumption that must be audited for each deployment.
4. We run the same PCRC protocol on three public ϕ-OTDR/DAS field corpora with different sensing geometries. The known-negative FAR follows the target level on measured recordings, held-out threat coverage reaches 0.87/0.68 on the two multichannel corpora, and three measured held-out nuisance families—BJTU watering, OptaSense longboard/gate motion, and Mendeley vehicles—are shown to pass the physics gate entirely, so that false-alarm control for them rests on verified-negative recalibration alone.
5. On a physics-motivated ϕ-OTDR simulation with five known and four unknown event classes, PCRC reduces the unknown-negative FAR from 0.42–0.64 for the classifier baseline to no observed automatic false alarms on the simulated inadmissible nuisance classes, while keeping known-threat recall at 0.998. We report these zero counts as finite-sample empirical observations, not as universal zero-risk claims, and also provide seed robustness, a two-dimensional convolutional neural network (2-D CNN) backbone, open-set detector comparisons, sensitivity to the gate threshold, and drift experiments.

Section 2 positions the work relative to DOFS recognition, open-set detection, conformal risk control, and false-alarm calibration. Section 3, Section 4 and Section 5 define PCRC and its conditional guarantees. Section 6 reports measured field evidence before the controlled assumption tests and deployment stress tests; Section 7 and Section 8 delimit the operating envelope and conclusions.

The public datasets used in the study are listed in the Data Availability Statement.

## 2. Related Work

### 2.1. Event Recognition in DOFS/DAS

A large body of work reports ϕ-OTDR/DAS event recognition with hand-crafted spectral and morphological features feeding support vector machine (SVM) or random forest (RF) classifiers, or with end-to-end one-dimensional and two-dimensional convolutional neural networks (1-D/2-D CNNs) and recurrent models operating on the space-time matrix [3,4,5,6]. Recent work further improves intrusion-recognition accuracy through two-dimensional time–frequency feature fusion [7] and wavelet packet decomposition–empirical mode decomposition (WPD–EMD) preprocessing with residual networks [8]. These studies mainly report closed-set accuracy and usually assume that test events belong to the training taxonomy. In field trials, false alarms from environmental and instrumental nuisances are often handled by post-filters or site-specific heuristics. The present work is complementary: it treats the recognizer as a component and adds a calibrated decision layer that uses both negative calibration data and simple physical checks.

### 2.2. Open-Set and Open-World Recognition

Open-set recognition rejects inputs from unknown classes [9,10,11], and open-world recognition additionally requires incremental incorporation of newly discovered classes [12]; out-of-distribution (OOD) detection scores novelty using maximum softmax probability (MSP) [13], temperature scaling with input perturbation [14], energy [15], or feature-space Mahalanobis distance [16]. These methods provide useful novelty scores, but a novelty score is not the same as a false-alarm budget, and it does not indicate whether a sample is physically plausible for a near-fiber disturbance. PCRC therefore uses the novelty score only for the review tier; automatic alarms are controlled by the calibrated threshold and the physics gate.

### 2.3. Conformal Prediction and Risk Control

Conformal prediction yields distribution-free, finite-sample coverage under exchangeability [17,18,19,20], with extensions to quantile regression [21] and to controlling general monotone risks (conformal risk control) [22]. Conformal *p*-values also support distribution-free outlier testing [23]. We use conformal calibration to set the alarm threshold on verified negatives. The limitation is that conformal validity is tied to the calibration distribution; if the negative distribution changes, the threshold must be recalibrated or supported by additional structure, as studied for covariate shift and for departures from exchangeability [24,25].

### 2.4. Physics-Informed Measurement

Physics-informed machine learning injects governing equations or invariants as priors or constraints [26,27,28]. In DOFS, several first-order signatures are known from the measurement physics: localized strain produces localized phase perturbations, genuine disturbances are not usually common-mode across all channels, and weak responses may be indistinguishable from the noise floor. We do not learn a surrogate partial differential equation (PDE) solver. Instead, we use these signatures as an interpretable consistency test.

### 2.5. Reliable Detection and False-Alarm Calibration

Controlling the false-alarm rate is a classical concern of measurement and detection. The Neyman–Pearson framework and constant-false-alarm-rate (CFAR) detectors set a decision threshold for a prescribed FAR, usually under a parametric noise/clutter model such as Gaussian or Weibull clutter [29,30,31]. Model mismatch is the main difficulty in open-world settings. The conformal layer of PCRC plays a related role without specifying a parametric noise law, provided that the verified calibration negatives and future gate-passing negatives are exchangeable. This is consistent with the metrological view that an alarm should be accompanied by an uncertainty or error-rate statement [32]. Recent neural network calibration work [33] improves in-distribution reliability, but it does not by itself address novel negative classes. The added element here is a DOFS-specific admissibility test that removes a subset of model-violating artifacts before the calibrated threshold is applied.

The individual ingredients used in PCRC—the classifier, logistic membership maps, geometric aggregation, maximum softmax novelty score, and conformal order statistic—are established techniques. PCRC is therefore a decision-layer contribution rather than a new feature extractor or classifier. The methodological contribution is their DOFS-specific integration into an interpretable physical-admissibility gate, calibration restricted to gate-passing verified negatives, a conditional FAR decomposition, and an alarm/review/discard workflow whose deployment assumptions can be audited explicitly.

## 3. Problem Formulation

Let $x\in X=R^{N_{z}\times N_{t}}$ be a decision window. Each window has a (latent) event-type label y∈Y and a binary operational threat indicator c(y)∈{0,1}. Here, c(y)=1 denotes an alarm-worthy intrusion, whereas c(y)=0 denotes a no-threat or negative event; *c* is distinct from the multiclass event type *y*. The type space partitions into known and unknown subsets, $Y=Y_{\mathrm{kn}}\cup Y_{\mathrm{unk}}$; only $Y_{\mathrm{kn}}$ is represented in training. A monitoring system emits a decision d(x)∈{ALARM,REVIEW,DISCARD}.

**Definition 1**  (Open-world alarm filtering)**.** *Given a user-specified false-alarm budget α∈(0,1), design d(·) so as to keep the auto-alarm rate on negatives (c=0), P{d(x)=ALARM∣c=0}≤α, as the negative distribution ranges over known* and *novel sources, while maximizing threat coverage P{d(x)∈{ALARM,REVIEW}∣c=1} over both known and unknown threats, at a bounded review load.*

The control over *arbitrary* novel negatives stated above is an idealized desideratum: Section 5 characterizes the structural assumptions (exchangeability and physical admissibility) under which it is provably attained, and Remark 3 makes explicit the regime (physically admissible novel nuisances) in which it must be relaxed.

**Evaluation strata.** We report metrics separately on four strata: known negative, unknown negative, known threat, and unknown threat. The distinction between known- and unknown-negative FAR is the crux: a method may satisfy the budget on the former while violating it arbitrarily on the latter.

**Remark 1**  (Why closed-set + conformal is not enough)**.** *Let s(x) be any score and let the alarm threshold λ be calibrated on negatives drawn from the in-distribution $P_{\mathrm{kn}}$. The resulting statement $P_{\mathrm{kn}}\left\{s>\lambda |c=0\right\}\leq \alpha$ is a statement about $P_{\mathrm{kn}}$. For a novel negative distribution Q with $Q\neq P_{\mathrm{kn}}$, no such bound follows.*

## 4. Method: Physically Consistent Risk Calibration

**Figure 1:**
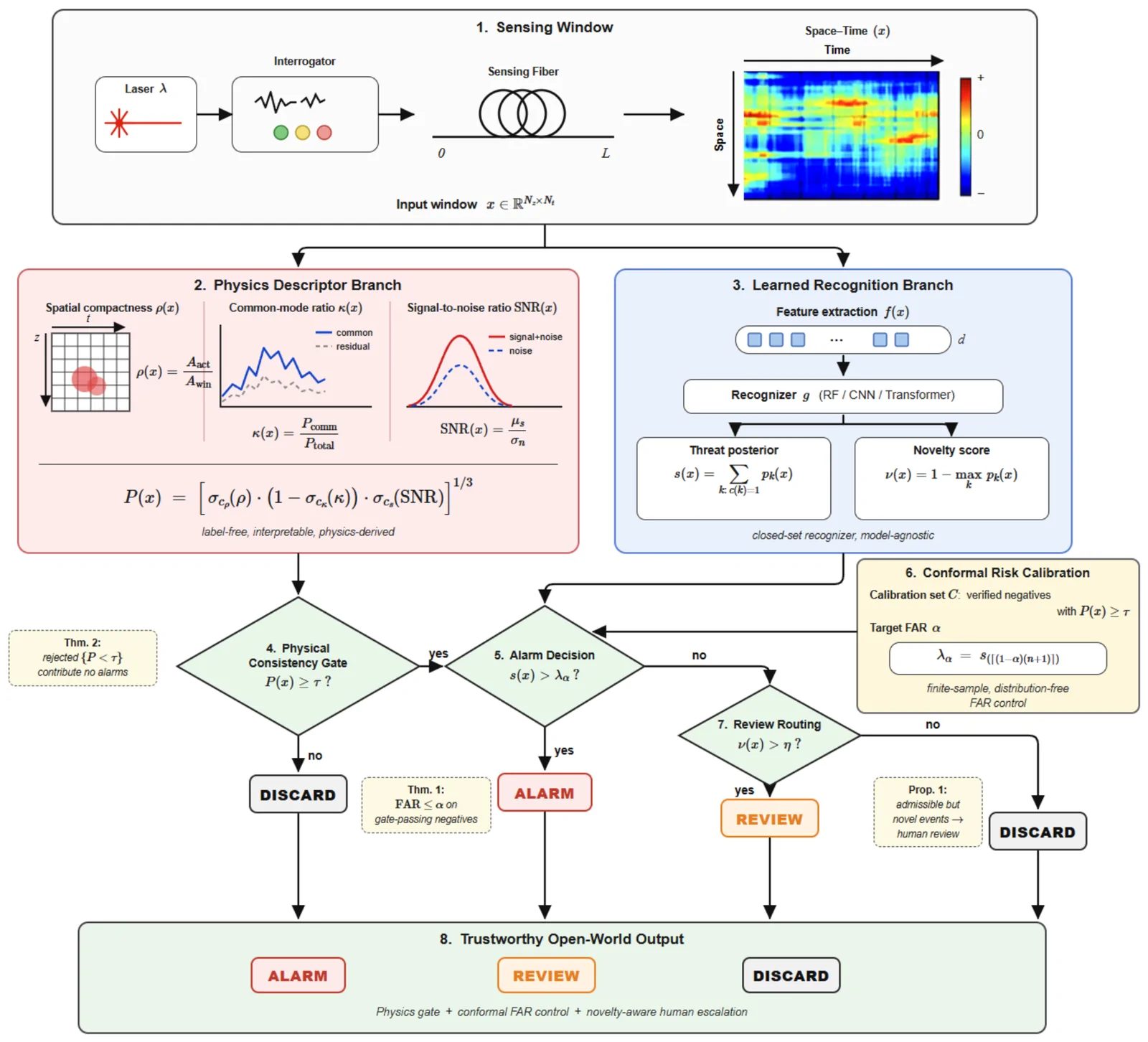
Overall architecture of Physically Consistent Risk Calibration (PCRC). **(1) Sensing:** A ϕ-OTDR/DAS interrogator produces a space-time window. **(2) Dual-path scoring:** A learned path feeds a recognizer *g* that emits a threat posterior s(x) and novelty score ν(x), while a parallel physics path maps spatial compactness ρ, common-mode ratio κ, and SNR to a consistency score P(x). **(3) Physics-gated risk calibration:** The gate P≥τ removes windows that are physically inadmissible under the stated descriptor model; for gate-passing windows the conformal threshold $\lambda_{\alpha }$, an order statistic of verified-negative scores, sets the automatic-alarm threshold. **(4) Three-tier decision:** Confident threats are alarmed, physically consistent but novel windows are sent to review, and the remaining windows are discarded.

Figure 1 summarizes PCRC. A window is described by (i) learned features feeding a closed-set recognizer that yields a threat posterior, and (ii) physics descriptors yielding a consistency score. A physics gate first rejects inadmissible windows; a conformal layer then sets the alarm threshold; finally, a three-tier rule decides between alarm, review, and discard.

### 4.1. Space–Time Representation and Recognizer

From *x* we compute the per-channel energy profile $e_{z}=\sum_{t}x_{z,t}^{2}$ and extract a compact feature vector f(x) comprising normalized band energies of the dominant channel, spectral centroid, and the physics descriptors below. A probabilistic recognizer $g:f\left(x\right)\mapsto \left(p_{1},\ldots ,p_{K}\right)$ outputs class posteriors over the *K* known types. The *threat posterior* aggregates the threat classes,

(1) $$s\left(x\right)=\sum_{k:\;c(k)=1}p_{k}\left(x\right)\in \left[0,1\right],$$

and the *novelty* score is $\nu \left(x\right)=1-\max_{k}p_{k}\left(x\right)$ (MSP). PCRC is agnostic to *g* (CNN, transformer, or, as here, a random forest).

### 4.2. Physical Consistency Score

We distill three complementary necessary, but not sufficient, admissibility conditions for a near-fiber mechanical disturbance. Spatial compactness tests localization of coupled energy; the common-mode ratio tests whether nearly identical traces appear across the array, as expected for some acquisition or electrical artifacts; and SNR tests separation from the local noise floor. These descriptors are transparent engineering checks, not a complete forward model of ϕ-OTDR physics.

**(1) Spatial compactness.** A localized source excites a compact set of channels. With $e_{\left(1\right)}\geq \cdots \geq e_{\left(N_{z}\right)}$ the sorted channel energies,

(2) $$\rho \left(x\right)=\frac{\sum_{i=1}^{m}e_{\left(i\right)}}{\sum_{i=1}^{N_{z}}e_{\left(i\right)}},\;m\ll N_{z},$$

is large for localized events and small for diffuse fields (wind/rain) or uniform artifacts.

**(2) Common-mode ratio.** Interferometric/electrical interference appears *identically* on all channels. With the common-mode trace $\bar{g}\left(t\right)=\frac{1}{N_{z}}\sum_{z}x_{z,t}$ and per-channel Pearson correlation $r_{z}=\mathrm{corr}\left(x_{z,\cdot },\bar{g}\right)$,

(3) $$\kappa \left(x\right)=\frac{1}{N_{z}}\sum_{z}r_{z}\in \left[-1,1\right]$$

is near 1 for common-mode artifacts and small for genuine localized events.

**(3) Signal-to-noise ratio.** With ${\bar{e}}_{\left(1:m\right)}=\frac{1}{m}\sum_{i=1}^{m}e_{\left(i\right)}$, the *mean* energy of the *m* dominant channels, the signal-to-noise ratio (SNR),

(4) $$\mathrm{SNR}\left(x\right)=10\log_{10}\;\left({\bar{e}}_{\left(1:m\right)}\;/{\mathrm{median}}_{z}e_{z}\right),$$

separates events from the noise floor. The median over all channels is used as the noise-floor estimate because it is insensitive to the localized excess energy that the descriptor is meant to detect. Note that the numerator is a mean and not a sum, so the descriptor and the membership center $c_{s}$ are independent of the footprint size *m*.

Mapping descriptor *u* through a smooth membership $\sigma_{c,w}\left(u\right)={\left[1+e^{-(u-c)/w}\right]}^{-1}$ makes both the engineering center *c* and transition width *w* explicit. Combining the three memberships by a geometric mean yields the *physical consistency score*:

(5) $$P\left(x\right)={\left[\sigma_{c_{\rho },s_{\rho }}\left(\rho \right)\cdot \left(1-\sigma_{c_{\kappa },s_{\kappa }}\left(\kappa \right)\right)\cdot \sigma_{c_{s},s_{s}}\left(\mathrm{SNR}\right)\right]}^{1/3}\in \left[0,1\right].$$

P(x) is high only when a window is *simultaneously* compact, non-common-mode, and above noise—i.e., admissible as a near-fiber mechanical disturbance. Crucially, *P* depends only on *x* and fixed physical thresholds; it requires no labels and is invariant to the threat taxonomy, so it transfers to unknown event types.

The logistic maps are deterministic monotone soft thresholds, not learned probabilities: each center marks an engineering transition and each width avoids an unstable hard step. The equal-weight geometric mean acts as a soft logical AND, so one physically implausible descriptor cannot be fully compensated by two large values, as it could under an arithmetic mean. This construction is not claimed to be a unique physical law; it is an auditable design whose descriptor margins must be checked during site commissioning. Table 1 summarizes the fixed, trained, and calibrated quantities used by PCRC.

### 4.3. Physics Gate and Conformal Risk Calibration

**Physics gate.** Define the admissibility gate G(x)=1[P(x)≥τ] for a physical-plausibility level τ.

**Conformal threshold.** Let $C={\left\{x_{i}\right\}}_{i=1}^{n}$ be a calibration set of verified *negatives*; here, c=0 again denotes a no-threat window under the binary threat indicator defined in Section 3. The calibration windows pass the gate and have scores $s_{i}=s\left(x_{i}\right)$. Let $s_{\left(1\right)}\leq \cdots \leq s_{\left(n\right)}$ and define the augmented value $s_{\left(n+1\right)}=+\infty$. For target FAR α, set

(6) $$\lambda_{\alpha }=s_{\left(k\right)},\;k=\left\lceil (1-\alpha )(n+1)\right\rceil ,$$

the augmented *k*-th order statistic (the conformal upper quantile). When k=n+1, $\lambda_{\alpha }=+\infty$ and no automatic alarm is issued; this conservative edge case avoids extrapolating above the largest observed calibration score.

A finite automatic-alarm threshold exists only when k≤n, which requires at least n≥⌈1/α⌉−1 verified, gate-passing calibration negatives (19 at α=0.05). This is a mathematical minimum, not a recommendation for statistical precision: deployment buffers should be larger and should reserve an independent audit subset for confidence-interval reporting. We note one implementation detail for reproducibility: in the released code, the k=n+1 case is clamped to the largest calibration score rather than to +∞. Clamping is anti-conservative and voids the finite-sample bound in that edge case; it should be replaced by the +∞ convention of Equation (6) in any deployment, and the buffer-size floor above should be enforced so that the case does not arise.

**Three-tier decision.** With a novelty threshold η (calibrated to a review budget),

(7) $$d\left(x\right)=\left\{\begin{array}{cc} D\mathrm{ISCARD}, & P(x)<\tau ,\\ A\mathrm{LARM}, & P\left(x\right)\geq \tau \wedge s\left(x\right)>\lambda_{\alpha },\\ R\mathrm{EVIEW}, & P\left(x\right)\geq \tau \wedge s\left(x\right)\leq \lambda_{\alpha }\wedge \nu \left(x\right)>\eta ,\\ D\mathrm{ISCARD}, & \mathrm{otherwise}. \end{array}\right.$$

Windows below the physics threshold are discarded before classification. Gate-passing windows are then compared with the conformal alarm threshold, and gate-passing but novel-looking windows are sent to review. Algorithm 1 summarizes the inference.
**Algorithm 1** PCRC inference for one window**Require:** window *x*; recognizer *g*; thresholds τ, $\lambda_{\alpha }$, η    compute physics descriptors ρ, κ, SNR and P(x) via Equation (5)    **if** 
P(x)<τ 
**then**           **return** Discard    **end if**    $\left(p_{1},\ldots ,p_{K}\right)\leftarrow g\left(f\left(x\right)\right)$    $s\leftarrow \sum_{c(k)=1}p_{k}$;   $\nu \leftarrow 1-\max_{k}p_{k}$    **if** 
$s>\lambda_{\alpha }$ 
**then**           **return** Alarm    **else if** 
ν>η 
**then**           **return** Review    **else**           **return** Discard    **end if**


## 5. Theoretical Analysis

We analyze the FAR and coverage properties of the decision rule. The proofs are provided immediately after the corresponding statements.

**Theorem 1**  (Finite-sample conformal FAR on plausible negatives)**.** *Assume the gated calibration negatives ${\left\{x_{i}\right\}}_{i=1}^{n}$ and a future gated test negative $x_{n+1}$ are exchangeable. With $\lambda_{\alpha }$ from Equation (6),*

(8) $$P\{s\left(x_{n+1}\right)>\lambda_{\alpha }|c=0,\;G=1\}\leq \alpha .$$

*The bound does not depend on the form of the score s or the recognizer g, but it does depend on exchangeability of the gated calibration and test negatives.*

**Proof.**  Condition on c=0, G=1. By exchangeability of $s_{1},\ldots ,s_{n},s_{n+1}$, the rank of $s_{n+1}$ among the n+1 scores is uniform on {1,…,n+1}. For k≤n, $P\left\{s_{n+1}>s_{\left(k\right)}\right\}\leq \left(n+1-k\right)/\left(n+1\right)$; for k=n+1, the augmented value $s_{\left(n+1\right)}=+\infty$ makes the probability zero. Substituting k=⌈(1−α)(n+1)⌉ therefore gives $P\{s_{n+1}>\lambda_{\alpha }\}\leq \alpha$ in both cases. □

**Theorem 2**  (Open-world FAR decomposition)**.** *Let negatives be drawn from an arbitrary mixture $Q=\pi \;Q_{G=1}+\left(1-\pi \right)\;Q_{G=0}$ of gate-passing and gate-failing negatives, with $\pi =P_{Q}\left\{G=1|c=0\right\}$. Under the decision of Equation (7),*

(9) $${\mathrm{FAR}}_{Q}=P_{Q}\left\{d=A\mathrm{LARM}|c=0\right\}=\pi \cdot \alpha_{Q_{G=1}},$$

*where $\alpha_{Q_{G=1}}=P_{Q_{G=1}}\left\{s>\lambda_{\alpha }\right\}$. Equation (9) is an exact decomposition rather than a bound; its content is not the algebra but the fact that the gate-failing mass contributes exactly zero, so that all FAR control reduces to the single conditional quantity $\alpha_{Q_{G=1}}$, which Theorem 1 bounds under exchangeability. Gate-failing negatives cannot trigger an automatic alarm under Equation (7). If, moreover, every novel negative is physically inadmissible, i.e., $\mathrm{supp}\left(Q_{\mathrm{novel}}\right)\subseteq \left\{x:P\left(x\right)<\tau \right\}$, then the novel-negative contribution to the automatic-alarm FAR is zero; if the remaining gate-passing negatives are exchangeable with C, then ${\mathrm{FAR}}_{Q}\leq \pi \alpha \leq \alpha$.*

**Proof.**  By Equation (7), d=ALARM⇒G=1. Thus $P_{Q}\left\{d=A\mathrm{LARM}|c=0\right\}=P_{Q}\left\{G=1|c=0\right\}\;P_{Q}\left\{s>\lambda_{\alpha }|c=0,G=1\right\}=\pi \;\alpha_{Q_{G=1}}$, which is Equation (9). Gate-failing negatives have G=0, hence never alarm, contributing zero irrespective of $Q_{G=0}$. If $\mathrm{supp}\left(Q_{\mathrm{novel}}\right)\subseteq \left\{P<\tau \right\}$, then all novel negatives are gate-failing (no false alarms); and if $Q_{G=1}$ is exchangeable with C, Theorem 1 gives $\alpha_{Q_{G=1}}\leq \alpha$, so the total is ≤πα≤α. □

**Remark 2.**  *Theorem 2 separates the two roles in PCRC. Conformal calibration controls FAR on the gate-passing negatives covered by the calibration set (Theorem 1). The physics gate removes windows in the structurally defined inadmissible set {P<τ} before they can be alarmed. Under the admissibility condition (A2) below, this is useful for nuisance sources such as common-mode interference or diffuse noise. Without that condition, conformal calibration alone must carry the FAR control and may need online recalibration.*

**Remark 3**  (Scope and assumptions)**.** *The statements above are conditional. **(A1) Exchangeability.** The gate-passing calibration and test negatives must be exchangeable. Under covariate shift, this assumption can fail unless $\lambda_{\alpha }$ is recalibrated from verified negatives collected on the live fiber (Section 6.3.8). **(A2) Physical admissibility of novel negatives.** The novel negatives must concentrate on the inadmissible set {P<τ}. This is an empirical assumption about a deployment, not a universal property of DOFS data. It is plausible for diffuse weather-like events and common-mode electrical or optical artifacts, but it fails for benign near-fiber mechanical events such as legitimate traffic, gate motion, or construction outside the protected zone. For such admissible novel nuisances, PCRC does not provide a zero-FAR statement; they are handled only by the conformal threshold if exchangeability is restored by recalibration, or by improvements to the recognizer.*

**Proposition 1**  (Threat coverage of the three-tier rule)**.** *For any threat x (c=1) with P(x)≥τ, d(x)∈{ALARM,REVIEW} whenever $s\left(x\right)>\lambda_{\alpha }$ or ν(x)>η. Consequently, the missed-threat event {d=DISCARD,c=1} requires the conjunction $\left\{P\geq \tau ,\;s\leq \lambda_{\alpha },\;\nu \leq \eta \right\}$. The review tier enlarges the set of surfaced threats without changing the automatic-alarm region.*

**Proof.**  Immediate from the cases of Equation (7): with P≥τ, Discard occurs only if $s\leq \lambda_{\alpha }$ and ν≤η; the complement $\left\{s>\lambda_{\alpha }\right\}\cup \left\{\nu >\eta \right\}$ yields Alarm or Review. The auto-alarm region $\left\{P\geq \tau ,\;s>\lambda_{\alpha }\right\}$ is unchanged by η, so the FAR is unaffected. □

**Proposition 2**  (Review-load–coverage trade-off)**.** *The novelty threshold η controls a monotone trade-off: lowering η weakly increases threat coverage and weakly increases the review rate P{d=REVIEW}. If η is calibrated on exchangeable gated, non-alarming known negatives with the same augmented quantile convention as Equation (6), the probability that an in-distribution negative is sent to review by the novelty rule is bounded by the selected budget β, up to finite-sample conformal conservatism. This workload statement is separate from the automatic-alarm FAR bound.*

**Complexity.** Beyond the recognizer forward pass, PCRC adds $O(N_{z}\log N_{z}+N_{z}N_{t})$ per window for the descriptors and O(1) for the gated thresholding.

## 6. Results

### 6.1. Evaluation Protocol Overview

We evaluate PCRC in two regimes. First, we run the protocol on three public ϕ-OTDR/DAS field corpora, so that the main calibration and coverage results are computed from measured recordings. No new field measurements were acquired for this study; interrogators, installations, and acquisition campaigns are documented in the corresponding source records [34,35,36]. We report only processing conditions under our control and do not infer hardware settings absent from the public metadata. Second, we use an in-house, physics-motivated synthetic testbed to control the four evaluation strata (known/unknown × threat/non-threat), physical admissibility, and domain drift. Sample-level binomial confidence intervals are reported where sample counts are available; repeated-seed experiments report means and standard deviations as stated in their captions.

Training, calibration, and testing are separated by role. Recognizer parameters and preprocessing statistics are learned only from known-class training data. The alarm threshold $\lambda_{\alpha }$ is fitted only from verified, gate-passing negatives in the calibration split, and η is fitted on known gated non-alarming calibration data. Held-out unknown classes are test-only in source-static experiments; they enter calibration only in experiments explicitly labeled as adaptive recalibration. Simulated windows are independent generated realizations and no realization is reused across roles.

For the public corpora the situation is weaker, and we state this limitation explicitly. The known-class windows of each corpus are split into training (50%), calibration (25%), and test (25%) roles by a *window-level* random permutation. Recording, session, and file identifiers are discarded during preprocessing and are not used to group windows, so adjacent windows originating from the same continuous recording can be assigned to different roles. For the OptaSense corpus this is not merely possible but certain: windows are cut with a length of 200 samples and a stride of 150, so consecutive windows overlap by 25% and overlapping pairs are split across roles. The BJTU and Mendeley corpora are split from window pools drawn from a small number of continuous acquisitions and are subject to the same concern without the explicit overlap. We therefore do not claim event-level independence for any public corpus. The consequence is that the known-class quantities—closed-set accuracy, known-threat recall ${\mathrm{Rec}}_{\mathrm{kn}}$, and, to a lesser extent, the known-negative FAR—are optimistic on the public corpora and must not be read as deployment generalization. The held-out-class quantities (${\mathrm{FAR}}_{\mathrm{nov}}$, ${\mathrm{Cov}}_{\mathrm{nov}}$, and the A2 audit) are unaffected by this mechanism, because entire classes are withheld from training and calibration; they carry the open-world conclusions of Section 6.2. Event-grouped resplitting with preserved provider identifiers is listed as required future work in Section 7.5. Because provider file names are available for the BJTU release, we have run a recording-grouped control on BJTU that bounds this optimism on at least one corpus. Loading the released files in their numeric order and splitting each known class into contiguous file-index blocks (first 50% training, next 25% calibration, last 25% test), so that temporally adjacent windows cannot cross roles, changes the known-class quantities in the expected direction: closed-set accuracy falls from 0.822±0.010 (window-level split, five seeds) to 0.738, and the known-negative FAR at α=0.05 rises from 0.030±0.027 to 0.111[0.055,0.212], above the nominal target—direct evidence that the window-level known-class numbers are optimistic and that exchangeability between calibration and test negatives is degraded by a temporal-block split. Known-threat recall did not decrease (0.979 under the grouped split vs. 0.921±0.047 under the window-level split), so the optimism is not uniform across quantities. The held-out-class results are unchanged: the watering A2 audit remains 0.000 and the novel-negative FAR remains high under both splits, confirming that the open-world conclusions do not depend on the split mechanism. Recording-grouped re-evaluation of the OptaSense and Mendeley corpora, whose released preprocessing discards the identifiers, remains future work.

### 6.2. Validation on Three Public DAS Field Datasets

#### 6.2.1. Datasets and Open-World Splits

We selected three corpora with different channel counts and event taxonomies (Table 2): (i) the Beijing Jiaotong University (BJTU) buried-fiber ϕ-OTDR set [34] ($N_{z}=12$ channels per window, six event types), (ii) a 1700-channel OptaSense perimeter DAS release [35] (spatially resampled to $N_{z}=160$, eight event types), and (iii) a single-channel Mendeley DAS set [36] (hammering vs. background vs. vehicle). For each corpus we form a leave-classes-out split: some classes are used as known classes for training and calibration, and the remaining classes are held out as unknown. The same descriptors, gate, conformal calibration, and three-tier rule are then applied to each corpus.

The public releases do not expose a uniform set of acquisition metadata. We therefore report the provider-stated geometry and channel count above, and refer readers to the source records for the interrogator and installation descriptions. The interrogator model, gauge length, native channel spacing, and event duration are not consistently available for all three processed releases and are not inferred here. Under our control, preprocessing statistics are fitted on the known-class training split, and unknown classes are never used to choose preprocessing, classifier, gate, or source-static calibration settings.

The processing chain applied to each corpus is as follows. OptaSense raw data are decimated by 20 in time (20 kHz → 1 kHz) and by 10 in space (1700 → 170 loci), DC-removed per channel, cut into 200-sample windows with a stride of 150, and interpolated to a fixed 160 channels, with at most 220 windows retained per recording. BJTU windows are transposed to 12×10,000, DC-removed per channel, and resampled to 1000 time samples. Mendeley windows are single-channel records of 2500 samples, capped at 5000 windows for each of the two known classes. Before the descriptors of Section 4 are computed, every *multichannel* window is additionally interpolated onto the common canonical grid $N_{z}=120$, $N_{t}=200$, so that *m*, the membership centers, and τ have the same meaning across corpora. This last step means that BJTU is *up*-sampled from 12 to 120 channels and OptaSense is down-sampled from 160 to 120. Up-sampling by interpolation does not create spatial information; it smooths the channel-energy profile and therefore biases the compactness descriptor ρ upward and the common-mode ratio κ upward relative to a natively 120-channel array. The BJTU physics-gate results should be read with this caveat: the upward bias of ρ raises the consistency score while the upward bias of κ lowers it, so the net effect on the gate is not determined a priori and must be measured. To quantify the bias, we recomputed P(x) on the native 12×1000 BJTU grid (footprint m=2 channels under the same one-eighth rule, memberships unchanged) and compared it with the canonical-grid values on the same windows. The gate decision is insensitive to the grid: the per-class gate-pass fractions agree to within 0.012 (all classes pass at 0.988 or higher on both grids), the watering A2 audit is 0.000 on both grids, and the known-class PCRC metrics are unchanged. The mean consistency score is in fact slightly lower on the canonical grid than on the native grid (e.g., watering 0.869 vs. 0.956; background 0.806 vs. 0.839), so canonical-grid resampling did not inflate gate passing on BJTU. The caveat that absolute descriptor values are not comparable across natively different channel counts stands, and a native-resolution descriptor variant for low-channel-count arrays remains future work. Of the eight semantic categories defined for the OptaSense release, seven are present in the processed corpus used here; the fibre-manipulation category was not available in the retrieved subset and is absent from all the reported results.

#### 6.2.2. Findings on Measured Fiber

**Figure 2:**
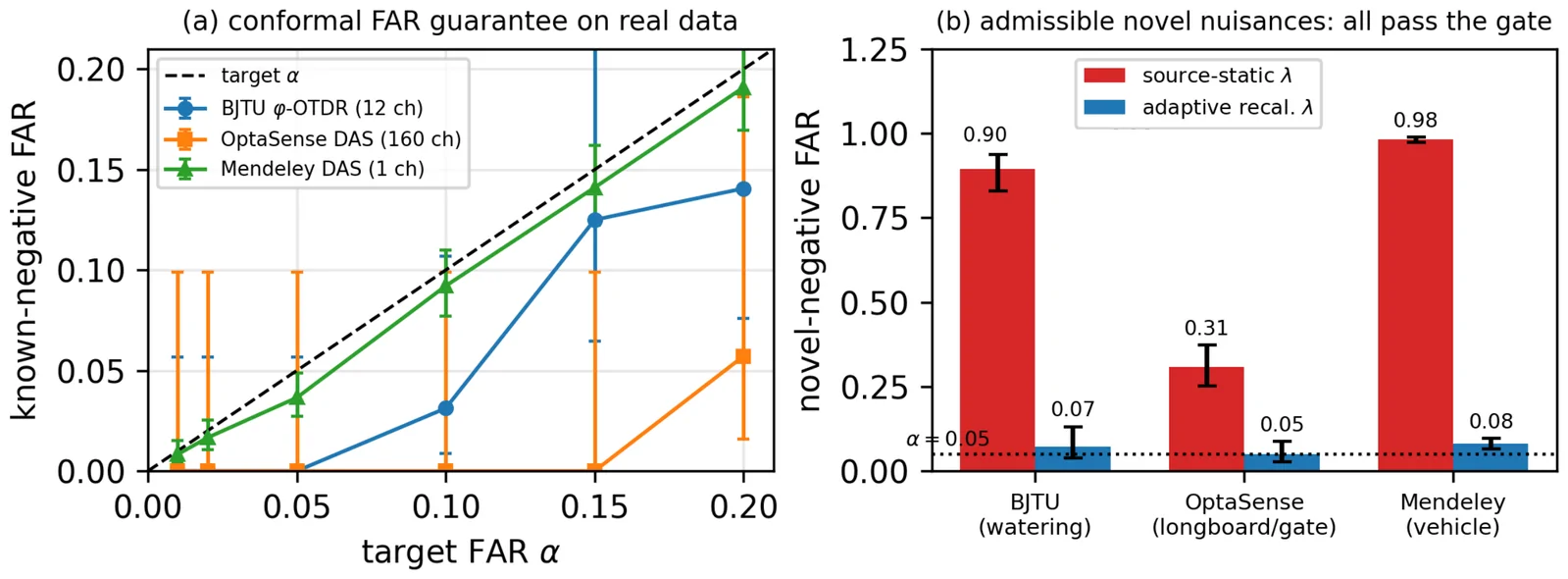
PCRC on three public DAS field datasets. (**a**) Known-negative FAR tracks the target across the three corpora. (**b**) All three measured held-out nuisance families are physically admissible and pass the gate (A2 rejection 0.000 in every case), so a source-static threshold violates the target badly; adaptive recalibration on verified novel negatives reduces the FAR to 0.072, 0.050, and 0.080, of which two remain above the nominal target. Error bars/CIs are Wilson 95%.

Table 3 and Figure 2 summarize the results at α=0.05.

On the known-negative classes, the empirical FAR stays at or below the target across the sweep (Figure 2a). At α=0.05, ${\mathrm{FAR}}_{\mathrm{kn}}=0.000\;\left[0,0.057\right]$ on BJTU, 0.000[0,0.099] on OptaSense, and 0.037[0.027,0.049] on Mendeley; known-threat recall is 0.866, 0.798, and 0.985, respectively. This is the part of the result directly covered by the conformal exchangeability assumption.

On the multichannel sets, the three-tier rule also surfaces held-out threat classes. At α=0.05, the auto-alarm recall of the held-out walking class is 0.836 on BJTU, and the review tier increases its total coverage to 0.868 with a negative review load of 0.041. On OptaSense, held-out threat coverage is 0.675.

The field data also show where the physics gate is insufficient on both multichannel corpora. On OptaSense, the held-out negatives are a longboard roll and a gate open/close. These compact, above-noise mechanical events are physically admissible; the A2 audit gives an inadmissible-rejection fraction of 0.000, and the source-static novel-negative FAR is 0.309[0.252,0.373]. On BJTU, the held-out watering class is a still more unfavorable admissible nuisance: a hose or sprinkler couples a localized, above-noise, non-common-mode disturbance into the buried fiber, which is exactly the signature the gate is designed to admit. The A2 audit again gives 0.000, and the source-static novel-negative FAR reaches 0.896[0.830,0.938]—the recognizer assigns most watering windows a threat posterior above a threshold calibrated on background alone. The single-channel Mendeley vehicle class is the third such case (${\mathrm{FAR}}_{\mathrm{nov}}=0.983\;\left[0.974,0.989\right]$ static), aggravated because no spatial gate is available. Adding verified examples of these nuisance families to the calibration buffer reduces the FAR to 0.050[0.028,0.087] on OptaSense, 0.072[0.038,0.131] on BJTU, and 0.080[0.067,0.097] on Mendeley.

Two of these three recalibrated values sit slightly above the nominal target. This is the expected behavior rather than a failure of the calibration: the verified buffer is finite (125 evaluation windows on BJTU), and folding a previously unseen nuisance family into an existing buffer restores exchangeability only to the extent that the family is represented in it. Taken together, the three corpora show that the gate contributes nothing against admissible nuisances—all three A2 audits are 0.000—and that the entire burden falls on verified-negative recalibration, which recovers approximately, but not exactly, the requested budget.

Two source-static novel-negative rates appear in this study and they are computed on different sample sets; we state the relationship explicitly rather than reporting a single reconciled number. The values quoted above and in the last two columns of Table 3 come from the recalibration experiment, in which the held-out novel negatives are partitioned at random into a verified half that is folded into the calibration buffer and a disjoint evaluation half on which both the static and the recalibrated FAR are measured. The evaluation half contains 220 windows on OptaSense, 125 on BJTU, and 1244 on Mendeley, which is what makes the static and recalibrated columns directly comparable. The α-sweep of Section 6.2 instead evaluates the static threshold on the *entire* novel-negative pool and gives 0.323[0.281,0.368] on 440 OptaSense windows, 0.888[0.843,0.921] on 250 BJTU windows, and 0.981[0.975,0.985] on 2487 Mendeley windows. The two pairs agree within their confidence intervals; the small differences are the finite-sample effect of halving the evaluation set, not a discrepancy between analyses. The latter remains above the nominal α=0.05 target, so we describe it as a substantial reduction rather than restoration of the target. These measured cases are counterexamples to extrapolating the controlled simulation’s zero observed alarms to all novel nuisances.

The single-channel Mendeley case is instructive: with no spatial descriptors, the physics gate is inapplicable and PCRC reduces to its conformal core, which makes the value of the gate on the multichannel corpora explicit by contrast. The descriptors remain individually interpretable on real data, with spatial compactness ρ and SNR the most discriminative single-feature separators of threat vs. non-threat (area under the receiver operating characteristic curve, AUROC: ρ=0.65/0.68, SNR =0.68/0.66 on BJTU/OptaSense).

### 6.3. Controlled Wave-Physics *ϕ*-OTDR Testbed

Because open-world evaluation requires controlled unknown classes, we complement field validation with an in-house, physics-motivated synthetic ϕ-OTDR testbed; it is not a commercial simulator and is not presented as a validated instrument-level digital twin. Its measurement principle follows the standard distributed-sensing principles summarized in Refs. [1,2]. The array has $N_{z}=120$ channels at ∆z=5 m and $N_{t}=200$ samples at $f_{s}=1$ kHz. A colored acquisition-noise floor is combined with localized Gaussian spatial envelopes, class-specific temporal signatures, inter-channel coupling, a moving envelope for vehicles, diffuse multi-center fields for weather-like events, and common-mode transients for electrical interference. Its purpose is to control the four evaluation strata and test the stated assumptions, not to replace field validation.

**Figure 3:**
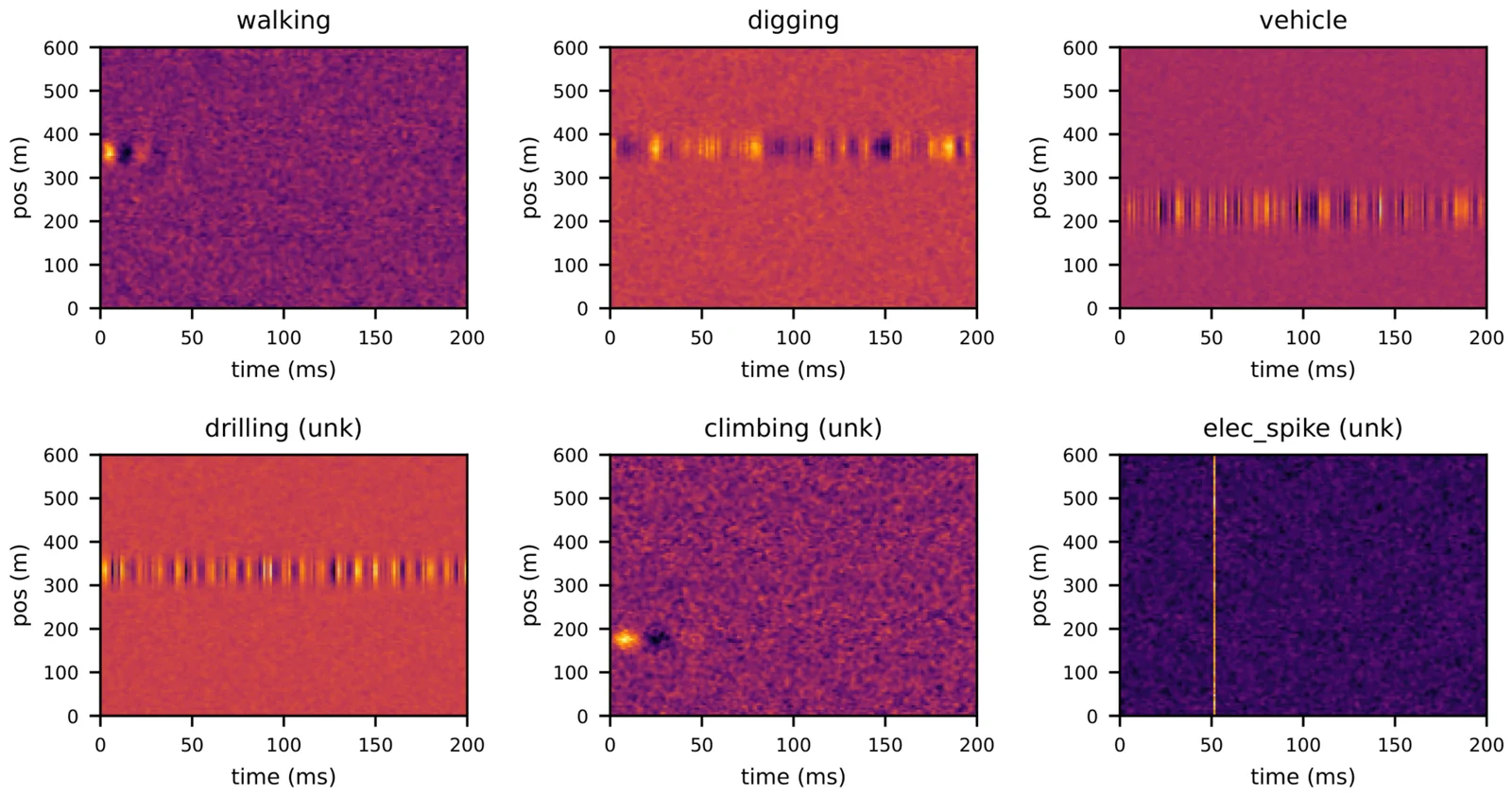
Representative space-time signatures (vertical: fiber position; horizontal: time; colors: relative signal amplitude within each panel, from low [dark purple] to high [yellow]). Known threats (walking, digging), a known non-threat (moving vehicle; note the diagonal streak), unknown threats (drilling, climbing), and an unknown non-threat (electrical spike, a vertical common-mode stripe). The electrical spike is physically inadmissible as a near-fiber disturbance and is rejected by the physics gate.

The corpus contains five known classes and four independent test-only unknown classes: threats (drilling, climbing) and non-threats (animal, electrical spike). We use 2600 training, 1400 calibration, and 2100 independently generated test windows. No realization is shared across roles. The recognizer is a 300-tree RF on f(x); feature-domain observation noise makes the closed-set task non-trivial. The fixed and calibrated settings are listed in Table 1. Per-class descriptor summaries are moved to Supplementary Table S1 to shorten the main manuscript. Representative space-time signatures from the controlled testbed are shown in Figure 3.

#### 6.3.1. Closed-Set and Open-Set Behavior

**Figure 4:**
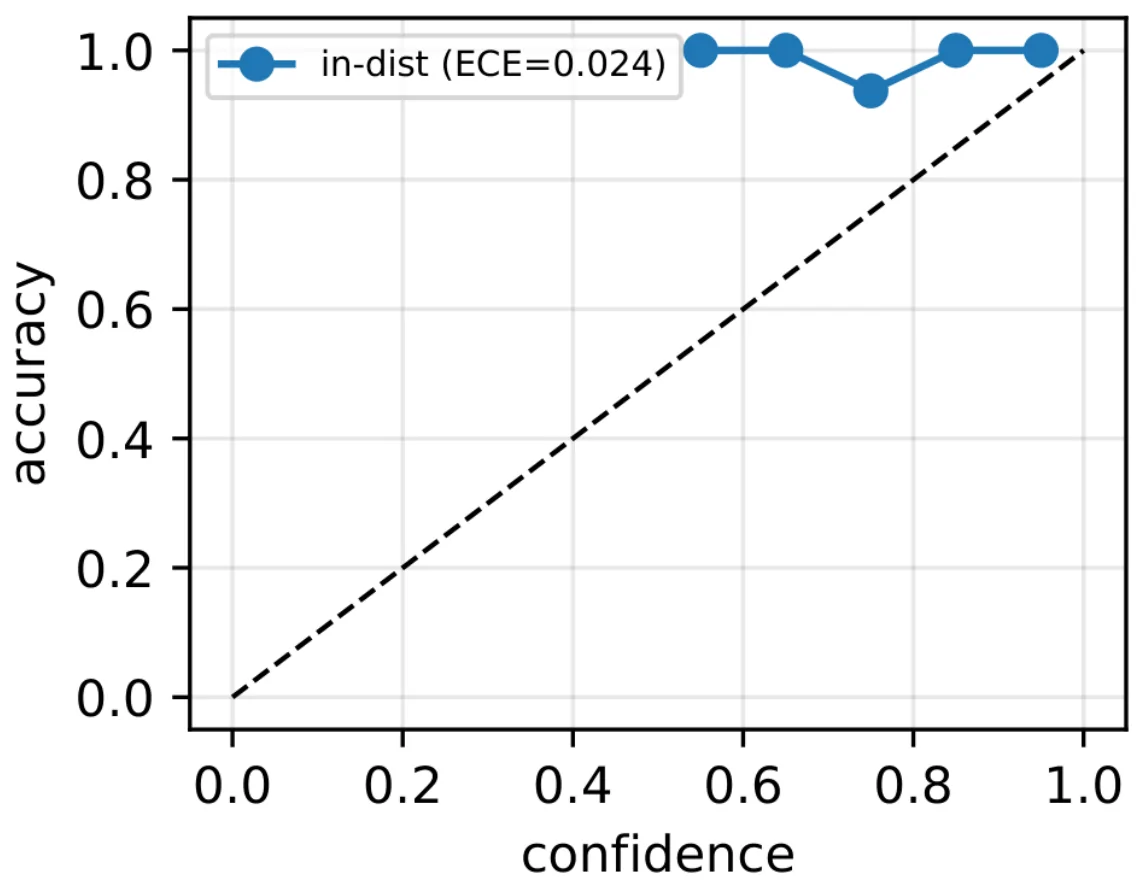
In-distribution reliability diagram of the recognizer (ECE = 0.024). The calibration curve is close to diagonal on known classes, but this does not imply FAR control on novel negative classes.

The recognizer attains a closed-set accuracy of 0.999 on known test classes with an in-distribution ECE of 0.024 (Figure 4). It is nevertheless overconfident on novel inputs: mean MSP is 0.975 on known classes versus 0.845 on unknown classes, giving an open-set AUROC of 0.75 for MSP. A class-wise confusion matrix, obtained from a paired seeded re-run that reproduces these aggregate metrics exactly, is reported in Supplementary Section S5: the known-class errors reduce to a single vehicle/digging confusion, whereas each held-out class collapses almost entirely onto one known class (drilling onto vehicle at a mean MSP of 0.911; climbing onto walking at 0.976). This gap motivates adding an explicit alarm-filtering layer instead of relying on the closed-set posterior alone.

#### 6.3.2. Open-World Alarm Filtering

**Figure 5:**
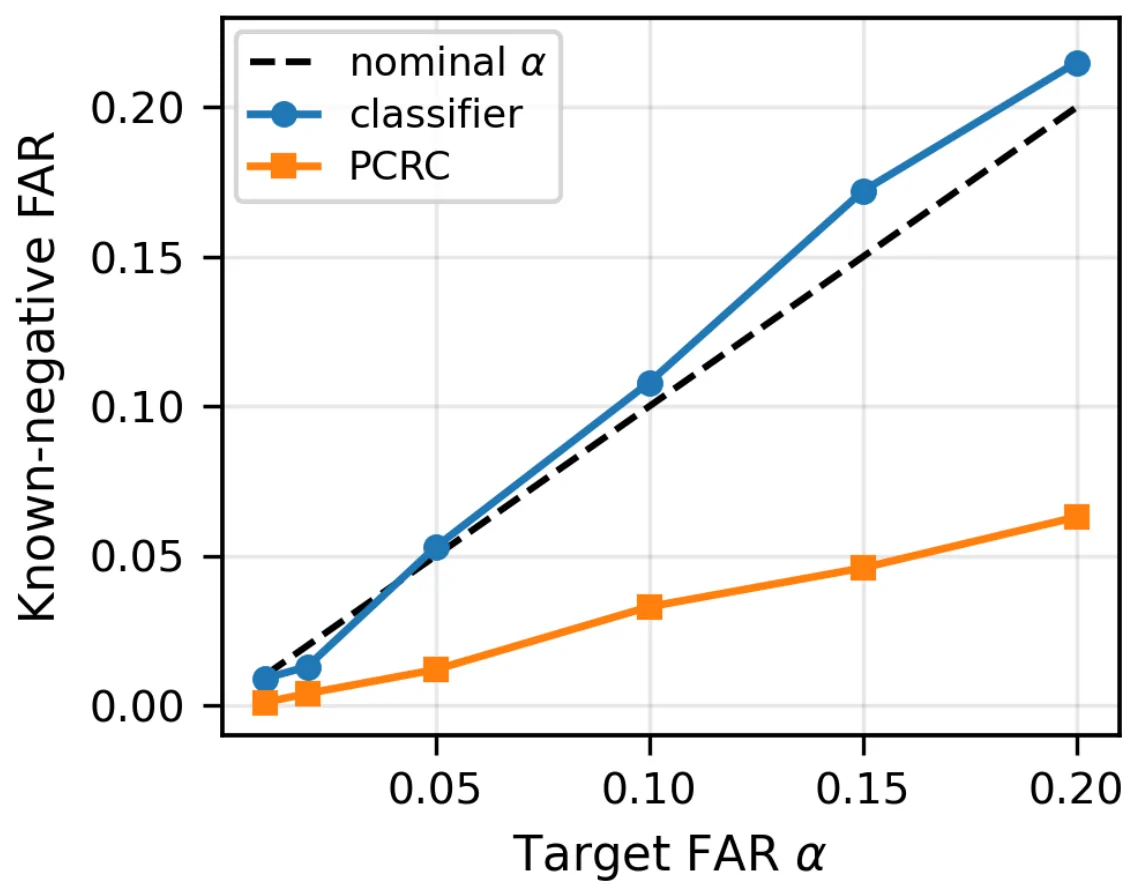
Empirical FAR on known negatives vs. target α. The rates track the target within finite-sample variation; individual empirical proportions can fall slightly above or below the nominal line.

**Figure 6:**
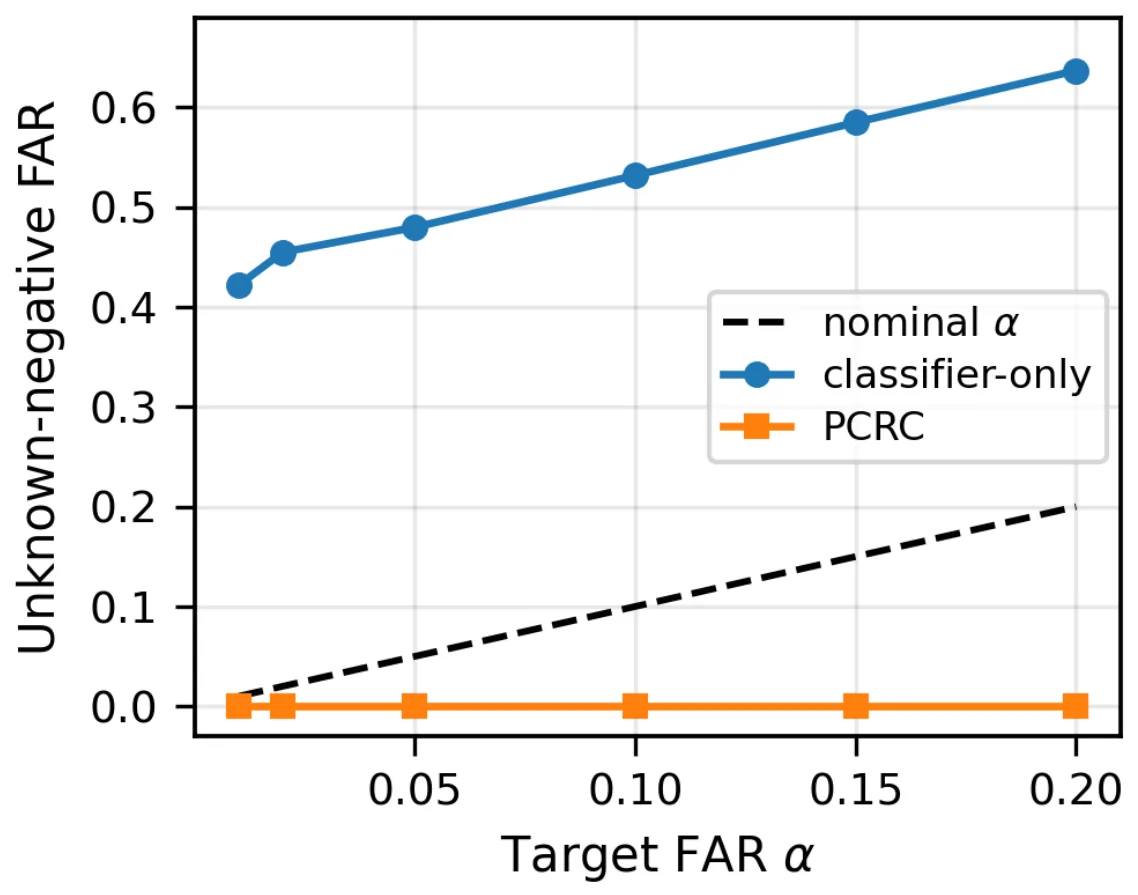
FAR on unknown negatives in the controlled simulation. The classifier baseline gives FAR values of 0.42–0.64; PCRC rejects the simulated inadmissible nuisance classes before the alarm threshold is applied.

Table 4 gives the main controlled simulation result. For the classifier-with-conformal baseline, the empirical FAR on known negatives follows the target α, but the FAR on unknown negatives is 0.42–0.64. PCRC removes the automatic alarms on the simulated unknown negatives across all tested α values, while keeping the known-negative FAR below α and preserving 0.998 recall on known threats. This result should be read together with the construction of the testbed: the unknown negatives are common-mode electrical spikes and diffuse events, so they satisfy the A2 admissibility assumption by design. Physically admissible novel nuisances require recalibration or recognizer improvements, as seen in the real-data results of Section 6.2. Figure 5 and Figure 6 show the known- and unknown-negative FAR curves.

Whenever a table reports ${\mathrm{FAR}}_{\mathrm{unk}}=0.000$ for PCRC, this denotes zero observed automatic false alarms in the finite test sample, not an exactly zero population risk. For $n_{\mathrm{unk}}$ evaluated unknown-negative windows with zero observed alarms, the one-sided 95% binomial upper confidence bound is $1-0.05^{1/n_{\mathrm{unk}}}$.

The price is a lower auto-alarm recall on unknown threats at strict α (e.g., 0.50 vs. 0.53 at α=0.01). The classifier baseline obtains slightly higher unknown-threat recall only while also alarming many unknown negatives. The review tier below is intended to recover part of this coverage without expanding the automatic-alarm region.

#### 6.3.3. Three-Tier Coverage

**Figure 7:**
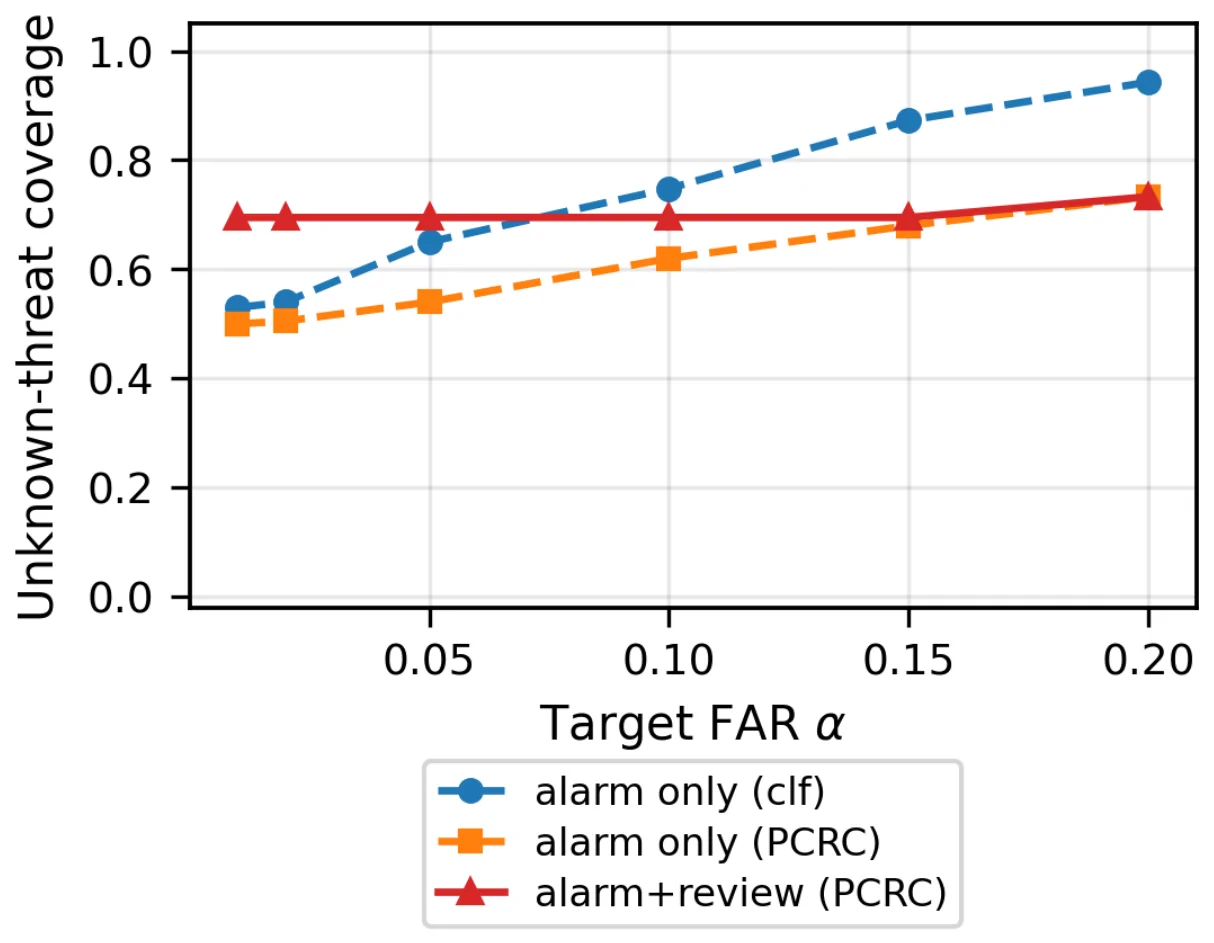
Unknown-threat coverage. Dashed curves show auto-alarm recall; the solid curve includes windows sent to review.

Table 5 reports the Alarm+Review coverage of the full three-tier rule. Routing physically consistent but novel windows to review raises unknown-threat coverage to 0.695–0.733 (from ≤0.54 for auto-alarm alone at small α) with a negative review load of 0.4–3.4%. Known-threat coverage remains at 0.998. Figure 7 contrasts auto-alarm recall with three-tier coverage.

#### 6.3.4. Ablation

Table 6 ablates the two ingredients at α=0.05. A fixed posterior threshold has no calibrated FAR target. Conformal-only calibration controls the known-negative stratum but gives 0.480 FAR on the simulated unknown negatives. The physics gate with a fixed threshold removes these inadmissible unknown negatives but does not set a user-specified FAR on gate-passing negatives. The full PCRC combines both pieces.

To isolate the physics-descriptor branch, we compare the fixed posterior decision at 0.5 with the identical decision preceded only by the physics gate. Adding the gate reduces the observed unknown-negative FAR from 0.025 to 0.000 and the overall negative FAR from 0.009 to 0.001, while known-threat recall decreases by 0.002 and unknown-threat recall is unchanged. Thus, the gate alone already removes the simulated diffuse/common-mode nuisance windows; conformal calibration serves the different role of setting a user-specified FAR target on gate-passing negatives.

#### 6.3.5. Backbone and Gate-Sensitivity Checks

To keep the main narrative focused, the detailed 2-D CNN table, five-seed table, and gate-threshold sweep are moved to Supplementary Sections S2–S4. In summary, the CNN reproduces the same qualitative failure of classifier-only conformal thresholding (unknown-negative FAR 0.63–0.96), whereas CNN+PCRC gives no observed automatic alarms on the simulated inadmissible nuisance classes. Across five RF seeds, PCRC reports 0.000±0.000 on those classes and a known-threat recall of at least 0.99. The post hoc τ sweep shows the expected trade-off: low τ admits inadmissible nuisances, while τ≳0.6 rejects weak threats. The interval 0.3≤τ≤0.55 is a simulation-specific plateau and is not used to claim a universal deployment threshold.

#### 6.3.6. Comparison with Open-Set Rejection Methods

A natural alternative to the physics gate is to reject novel inputs with an open-set/OOD detector. We compare maximum softmax probability (MSP) [13], Energy [15], OpenMax [10], and Mahalanobis distance [16] on the same CNN backbone, threat score, verified-negative calibration data, and target α=0.05. Every method uses $A\mathrm{LARM}=\mathrm{accept}\left(x\right)\wedge [s\left(x\right)>\lambda_{\alpha }]$, with $\lambda_{\alpha }$ recalibrated on its accepted known negatives. The OOD thresholds retain 90% of the least-novel known-calibration inputs (a high true-positive-rate, TPR, operating point), whereas PCRC uses its physics-defined τ=0.4. The comparison is therefore not exactly acceptance- or recall-matched. We report both FAR strata and both threat-recall strata so this operating-point difference is visible, and use Table 7 only as a transparent common-backbone comparison, not a universal ranking. A fully matched Pareto sweep requires the retained sample-level scores and is not reconstructed from summary tables.

**Figure 8:**
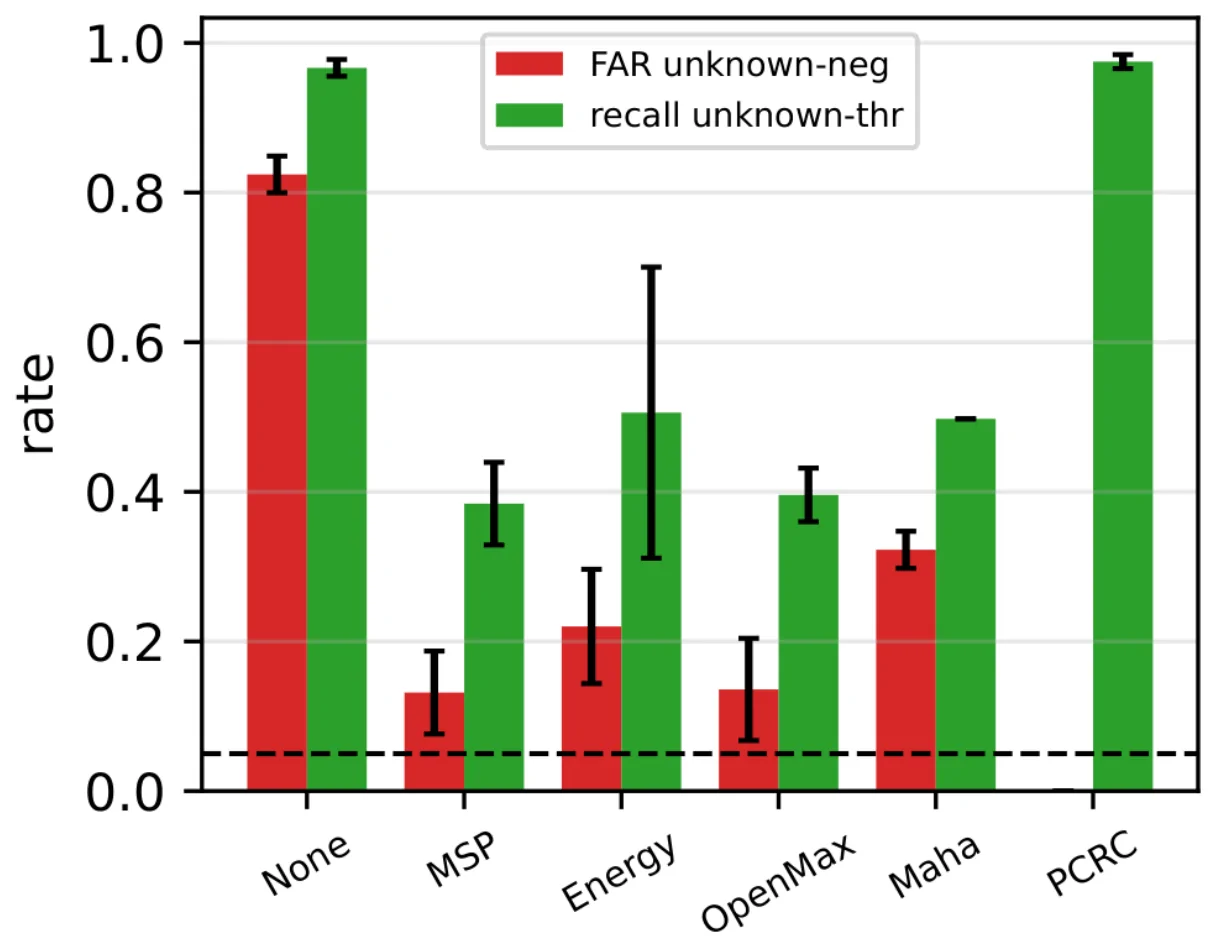
PCRC vs. open-set rejection (α=0.05, mean ± std, three seeds). Open-set detectors reduce unknown-negative FAR but also reduce unknown-threat recall. Dashed line: target α.

The open-set baselines achieve higher novelty-detection AUROC than the physics score (0.73–0.88 vs. 0.51) and reduce the unknown-negative FAR to 0.13–0.32. However, they also reject many unknown threats, so unknown-threat recall falls to 0.38–0.51; known-threat recall also falls to 0.83–0.87. PCRC has near-chance known-vs-unknown AUROC because the physics score is not designed as a novelty detector. In this experiment that is beneficial: it retains physically plausible unknown threats while rejecting the inadmissible nuisance classes. Figure 8 visualizes this common-backbone comparison.

One number in Table 7 requires comment, because it does not carry over to the primary experiment. PCRC attains ${\mathrm{Rec}}_{\mathrm{unk}}=0.975$ here, whereas the random forest protocol of Table 4 attains only 0.540 at the same α. The gate is identical in both cases and passes the same unknown threats; the difference is entirely in the threat posterior s(x) supplied by the backbone. The 2-D CNN operates on the pooled space-time window and assigns unknown drilling and climbing windows a threat posterior above the calibrated threshold, whereas the random forest operates on the compact feature vector f(x) with added observation noise and places roughly half of those windows below it. The comparison in Table 7 is therefore a statement about rejection rules at a fixed backbone, not about the unknown-threat recall that PCRC should be expected to deliver; the more conservative random forest figures of Table 4 are the ones carried into the Discussion, and the review tier exists precisely because auto-alarm recall on unknown threats is backbone-dependent. Thus, for alarm filtering, the key question is not only whether an input is novel, but whether it is physically admissible as a near-fiber disturbance.

#### 6.3.7. Field-Validation Protocol and Sim-to-Real Transfer

Because the simulated strata are controlled, we also examine how calibration behaves under a shift between the source fiber and a deployment fiber. We describe a field-validation protocol and then run a synthetic sim-to-real stress test.

**Protocol.** A perimeter deployment can proceed in three stages. (1) *Bootstrap*: Train the recognizer offline on a labeled source corpus (public ϕ-OTDR/DAS sets such as those in Section 6.2, or a vendor pilot), and fix the physical descriptors and initial gate τ. (2) *Online recalibration*: Recompute the conformal threshold $\lambda_{\alpha }$ from a rolling buffer of verified no-threat windows on the live fiber. (3) *Audit*: Report empirical FAR versus target α with binomial confidence intervals on held-out verified negatives, and track unknown-threat coverage using controlled test events where possible.

**Figure 9:**
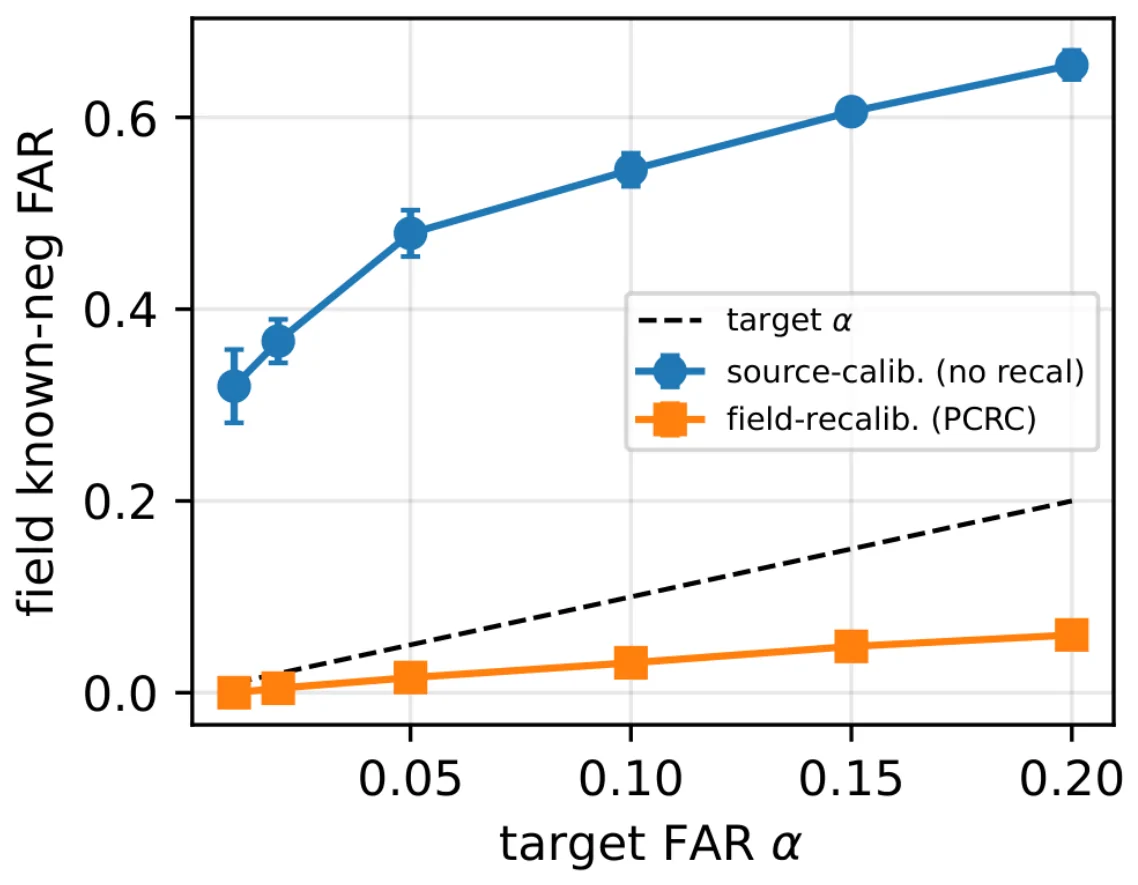
Empirical known-negative FAR versus target α under sim-to-real shift (mean ± std, three seeds). The source-calibrated threshold violates the target on the shifted fiber; recalibration on verified field negatives restores the target-level FAR. Dashed line: target α.

**Sim-to-real stress test.** We mimic a noisier, differently coupled field fiber by raising the noise floor (0.9→1.25), the inter-channel coupling (0.30→0.50), and the interference amplitude (1.0→1.3) while attenuating mechanical coupling (1.0→0.82). The recognizer is trained on the *source* domain only and tested on the *field* domain; “SRC” uses the source-calibrated $\lambda_{\alpha }$, “FLD” applies the online recalibration of stage (2). The results (mean over three seeds, α=0.05) are given in Table 8 and Figure 9.

**Figure 10:**
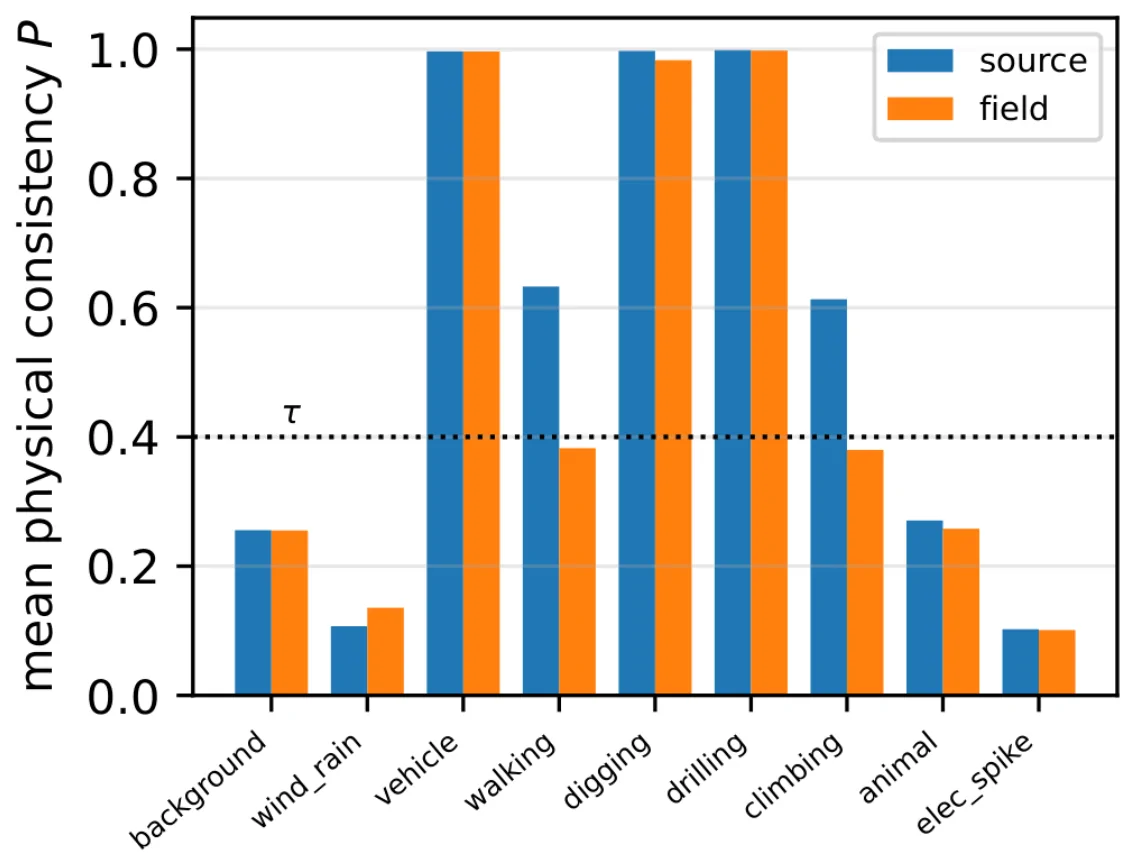
Per-class physical-consistency score *P* in the source versus field domain. Low-energy threats (walking, climbing) degrade as the field noise floor erodes their SNR, motivating field checks of τ.

The per-class physical-consistency scores under the same shift are compared in Figure 10.

The source-calibrated threshold does not transfer well to the shifted fiber: the classifier has ${\mathrm{FAR}}_{\mathrm{kn}}=0.479$, and PCRC still has ${\mathrm{FAR}}_{\mathrm{kn}}=0.146$. Recalibration on verified field negatives reduces these values to 0.049 and 0.016, respectively. The physics gate continues to reject the simulated unknown nuisance classes because their descriptor patterns remain diffuse or common-mode under the shift. The exception is weak threats: walking and climbing fall from P≈0.63/0.61 to 0.38/0.38 as the field noise floor erodes their SNR, dropping below τ=0.4 and reducing PCRC’s field known-threat recall to 0.66. This suggests that both $\lambda_{\alpha }$ and, for low-energy intrusions, τ should be checked on the deployment fiber.

#### 6.3.8. Deployable Online Recalibration Under Drift

**Figure 11:**
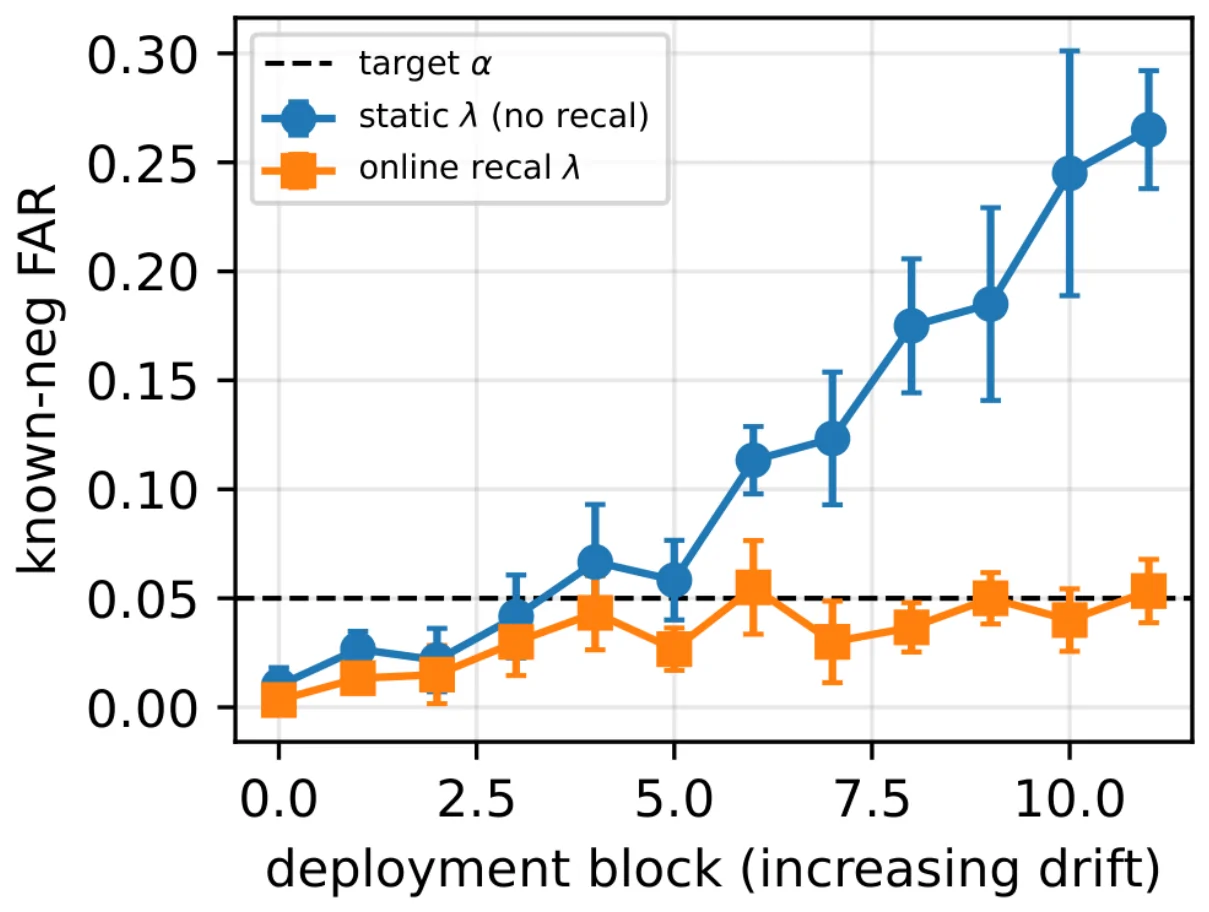
Known-negative FAR across twelve deployment blocks of increasing drift (mean ± std, five seeds). A static source-calibrated threshold drifts above the target α; sliding-window recalibration on verified negatives keeps the empirical FAR near the target.

A live perimeter is non-stationary: the noise floor, coupling, and interference gains drift over hours to seasons. To quantify what a deployable PCRC must achieve, we drive the wave-physics generator through a slowly drifting acquisition regime (the noise floor rises 0.9→1.45, inter-channel coupling 0.30→0.55, and interference gain 1.0→1.45, while mechanical coupling falls 1.0→0.80 across twelve deployment blocks) and compare a static source-calibrated λ against sliding-window recalibration on a rolling buffer of verified negatives (physics gate τ held fixed). All rates are pooled over five seeds with Wilson 95% intervals (Table 9, Figure 11).

Under drift, the static threshold lets the known-negative FAR climb to 0.111 [0.104,0.118], above the target α=0.05. Sliding-window recalibration reduces it to 0.033 [0.029,0.037] without retraining the recognizer. The novel-negative FAR remains at 0.000 [0.000,0.001] because the simulated novel nuisance windows remain below the physics threshold throughout the drift. The online system gives a strong-threat recall of 0.585 [0.570,0.600] and a weak-threat recall of 0.618 [0.604,0.633] at a negative review load of 0.095 [0.090,0.101]. The static-arm routing and review cells of Table 9 were obtained by a paired re-run of the fully seeded drift experiment: the identical random stream reproduces every previously reported value exactly (both FAR strata and the online recall and review figures), so the static and online columns are computed on the same realizations and are directly comparable. The comparison makes the recalibration trade-off explicit: sliding-window recalibration restores the known-negative FAR from 0.111 to 0.033, but raises the strong-threat missed rate from 0.341 to 0.415 and the negative review load from 0.043 to 0.095; weak-threat routing is unchanged because the weak-threat scores that pass the gate lie above both thresholds.

#### 6.3.9. Physical Interpretability of the Gate

**Figure 12:**
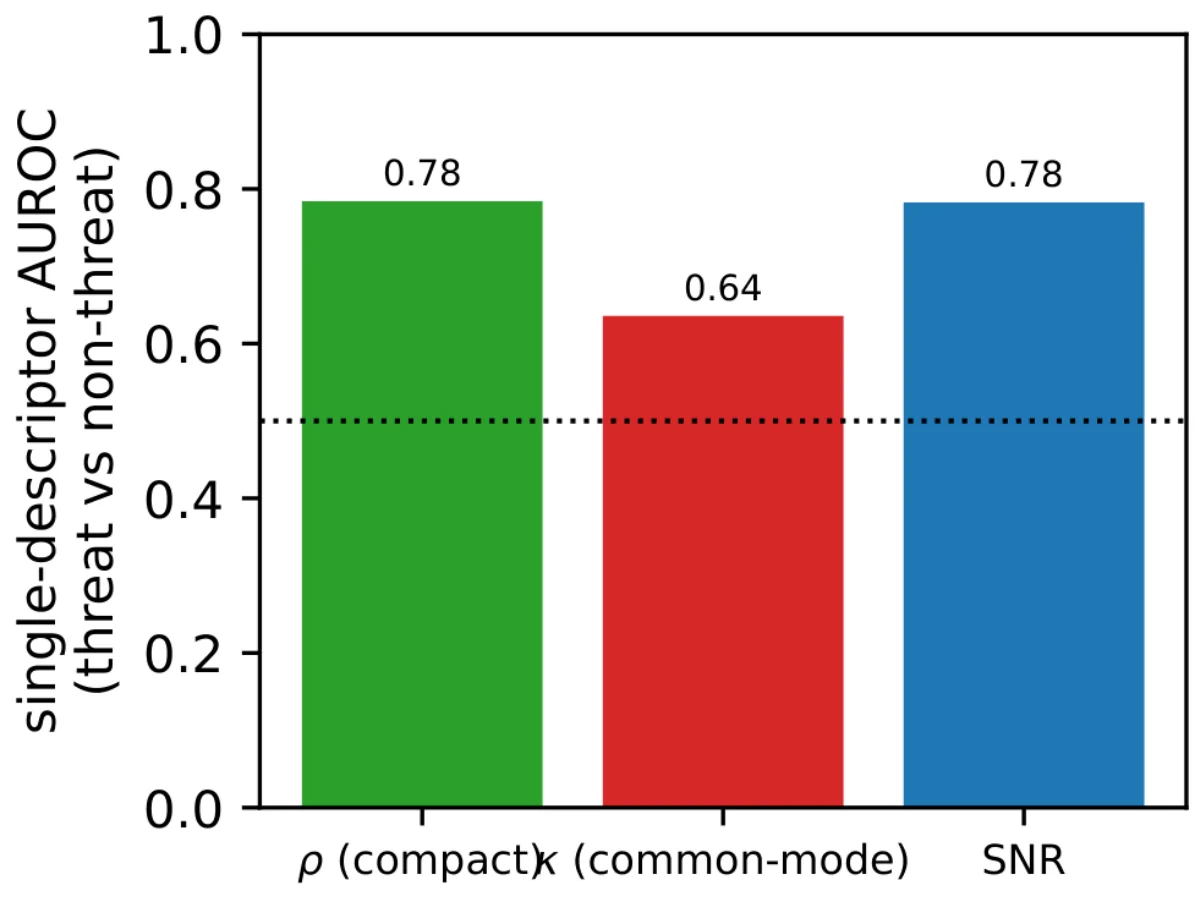
Single-descriptor separability (AUROC, threat vs. non-threat). The dotted line is chance.

Because the gate is a closed-form function of three physical descriptors, its decisions can be inspected after the fact. Treated as a single feature, spatial compactness ρ separates threats from non-threats at an AUROC of 0.784, SNR at 0.783, and the common-mode ratio κ at 0.636 (Figure 12); κ separates electrical common-mode spikes from localized disturbances at an AUROC of 0.807. For threat-positive AUROC, the common-mode descriptor is direction-adjusted as 1−κ, because large raw κ denotes a nuisance-like common-mode response. An operator can therefore identify whether insufficient spatial localization, common-mode structure, or low SNR caused a window to be rejected.

## 7. Discussion

### 7.1. What the Guarantee Does and Does Not Cover

The physics gate is useful when nuisance or artifact sources are physically inadmissible under the descriptor model, for example, diffuse fields or common-mode interference. It is not intended to reject benign events but fiber-coupled mechanical events such as nearby traffic or gate motion. Those cases must be represented in the calibration buffer or handled by the recognizer. Theorem 2 therefore applies only to the portion of the negative distribution that the gate rejects; physically admissible novel nuisances remain part of the conformal calibration problem.

### 7.2. The Measured Cost of Admissible Novel Nuisances

The field results of Section 6.2 quantify this limitation on all three corpora, and the pattern is uniform. On the OptaSense perimeter corpus, the held-out longboard roll and gate open/close are compact, above-noise mechanical events; the A2 audit gives an inadmissible-rejection fraction of 0.000, and a source-static threshold yields a novel-negative FAR of 0.309[0.252,0.373]. On the BJTU buried-fiber corpus, the held-out watering class is admissible for the same reason and is the most damaging case on multichannel data: A2 rejection is again 0.000 and the static novel-negative FAR is 0.896[0.830,0.938]. On the single-channel Mendeley corpus, the held-out vehicle class produces a static novel-negative FAR of 0.983, and no spatial descriptor is available. In all three cases, adding verified novel negatives to the calibration buffer reduces the FAR to 0.050[0.028,0.087], 0.072[0.038,0.131], and 0.080[0.067,0.097], respectively—substantial reductions, but two of the three remain above the nominal α=0.05. The consistency of the three A2 audits at exactly 0.000 is the central negative result of this study: on measured fiber, the physics gate contributed nothing against any nuisance family that was mechanically coupled to the cable, and its demonstrated value is confined to the diffuse and common-mode artifacts of the controlled testbed. The sim-to-real stress test shows a second limitation: weak-threat SNR can fall below the fixed gate threshold, reducing recall unless τ is checked on the deployment fiber. These results delimit the operating envelope of PCRC: rejected inadmissible mass is handled by the gate, while admissible nuisance mass is governed by recalibration and recognition quality.

### 7.3. False Alarms, Missed Threats, and Review Workload

Recalibration is not cost-free. In the sim-to-real comparison of Table 8, field recalibration lowers the classifier known-/unknown-negative FAR from 0.479/0.882 to 0.049/0.078, while the known-/unknown-threat recall falls from 1.000/0.737 to 0.910/0.433; the corresponding missed-threat rates rise from 0.000/0.263 to 0.090/0.567. For PCRC, the known-negative FAR falls from 0.146 to 0.016 and the unknown-negative FAR remains at zero observed alarms for the constructed inadmissible nuisances, while the known-/unknown-threat recall changes from 0.698/0.283 to 0.660/0.160 (missed rates 0.302/0.717 to 0.340/0.840). In the twelve-block drift test, online recalibration lowers the known-negative FAR from 0.111 to 0.033 and yields strong-/weak-threat missed rates of 0.415/0.382 at a negative review load of 0.095. The paired static arm, obtained from the seeded re-run described in Section 6.3.8, misses fewer strong threats (0.341 versus 0.415) at the same weak-threat missed rate (0.382) and less than half the negative review load (0.043 versus 0.095), so recalibration purchases the FAR reduction with additional strong-threat misses and review workload. Consequently, the study supports a joint FAR–recall–review trade-off, not the claim that recalibration improves every metric simultaneously.

### 7.4. Operational Recommendations

These observations lead to the following commissioning checklist:

- *Independent negative verification.* Admit a window to calibration only when evidence independent of the classifier—for example, synchronized video, access-control and maintenance logs, or an operator-confirmed quiet interval—supports the no-threat label. Model confidence alone is not verification.
- *Quarantine, calibration, and audit buffers.* Place newly verified windows in quarantine, screen them for duplication and threat contamination, and then divide them into disjoint rolling calibration and audit buffers. For a finite threshold, the gate-passing calibration buffer must contain at least ⌈1/α⌉−1 windows; substantially more are needed for a useful held-out confidence interval.
- *Commissioning (A2) audit.* Before relying on the gate, measure both the inadmissible-rejection and gate-pass fractions of representative site-specific nuisances, including weather, interference, and legitimate mechanical activity. Nuisance families with a high gate-pass fraction belong in verified-negative calibration.
- *Triggered recalibration and rollback.* Recompute $\lambda_{\alpha }$ when the audit-buffer FAR interval exceeds the budget, descriptor or score distributions drift beyond a site-defined tolerance, sensing hardware/coupling changes, or a newly verified nuisance family appears. Retain the previous threshold and buffer snapshot; reject the update and roll back if the independent audit FAR or controlled-event recall worsens beyond the deployment acceptance limits.
- *Contamination control.* Keep threat drills and unresolved windows out of the negative buffer, cap repeated windows from one episode, preserve event/session identifiers, and require secondary review for samples near the gate or alarm boundary. A suspected contamination event invalidates the affected calibration epoch.
- *Site-adaptive gate level.* On deployments dominated by low-energy intrusions (walking-type threats over lossy soil), verify the weak-threat margin *P* against τ and, if needed, lower τ within the safe plateau or use an SNR-adaptive membership width.
- *Single-channel deployments.* Where no spatial diversity exists, the gate is inapplicable; plan review capacity and recalibration cadence accordingly, since only the conformal core applies.
- *Continuous audit and workload.* Track FAR, threat recall/missed-threat rate, review fraction, absolute reviews per operating hour, and review latency together. Probe unknown-threat coverage with controlled test events where possible; an FAR reduction is unacceptable if missed threats or operator workload exceed the site’s limits.

### 7.5. Limitations and Future Work

The consistency thresholds in Equation (5) are physically interpretable but fixed in the present study; adapting them under conformal risk constraints is future work. The cleanest separation of known/unknown and threat/non-threat strata comes from the synthetic testbed. Both multichannel corpora now supply a measured held-out novel-negative stratum, but each contributes only one or two nuisance families, and the BJTU stratum depends on the labeling convention for the watering class recorded in Table 2. The public-corpus splits are window-level rather than event-level: recording identifiers are discarded in preprocessing, and the OptaSense windows additionally overlap by 25%, so known-class recall and closed-set accuracy on the public corpora are optimistic and are not used as evidence of deployment generalization. The held-out-class results are not affected by this mechanism. The recording-grouped BJTU control of Section 6.1 quantifies the optimism on one corpus (closed-set accuracy falling by 0.084, known-negative FAR rising to 0.111, above the target) while leaving the open-world strata unchanged; repeating this control on OptaSense and Mendeley, whose released preprocessing discards the identifiers, remains required future work. A second methodological limitation is the canonical-grid resampling: interpolating the 12-channel BJTU array up to 120 channels smooths the channel-energy profile and biases the compactness and common-mode descriptors; the native-grid sensitivity check of Section 6.2 shows that the gate decision and A2 audit on BJTU are insensitive to this resampling, though absolute descriptor values remain grid-dependent. The testbed confusion matrix has been recovered by a paired seeded re-run (Supplementary Section S5); the retained aggregate outputs still do not permit reconstruction of acceptance-matched baseline curves. Finally, no longitudinal live deployment with an operator-in-the-loop review queue was conducted. Future work should preserve event identifiers and raw scores, report class-wise confusion/support and matched FAR–recall–acceptance curves, add propagation-speed and multi-window-persistence descriptors, and measure reviews per hour and review latency in a prospective deployment.

## 8. Conclusions

We studied open-world alarm filtering for distributed optical fiber sensing and introduced Physically Consistent Risk Calibration (PCRC), which combines a label-free physical-admissibility gate, an augmented conformal alarm threshold, and an alarm/review/discard rule. Measured field results show that the known-negative FAR can track the target under exchangeability, while every physically admissible novel nuisance we could measure—watering, gate motion, longboard roll, and vehicle vibration—passed the gate untouched, with an inadmissible-rejection fraction of 0.000 on all three corpora, and required verified-negative recalibration that recovered the requested budget only approximately. In particular, on single-channel deployments the spatial gate is unavailable and PCRC reduces to its conformal core: on the Mendeley corpus the recalibrated novel-negative FAR remained at 0.080[0.067,0.097], above the nominal α=0.05, so single-channel operation is an explicit boundary of the method—the target FAR on admissible novel nuisances is not recoverable there without either spatial diversity or a stronger recognizer. The controlled synthetic testbed shows zero observed automatic alarms on nuisance classes constructed to violate the admissibility conditions, but this finite-sample observation is neither a universal zero-risk result nor evidence that all field nuisances satisfy A2. Recalibration can also trade lower FAR for higher missed-threat rate and review workload. PCRC should therefore be interpreted as an auditable conditional decision layer: deployment requires independently verified negatives, event-level data governance, site-specific gate checks, drift-triggered recalibration, and joint monitoring of FAR, recall, and human review load.

## Figures and Tables

**Table 1:** Fixed, trained, and calibrated quantities used by PCRC. The membership centers and widths below are identical in the controlled testbed and in all field-corpus runs: every multichannel window is first interpolated onto a canonical 120×200 ($N_{z}\times N_{t}$) grid, so the descriptor definitions and the spatial footprint rule are shared across corpora. The spatial gate is disabled for the single-channel corpus.

Quantity	Value/Rule	Source Role	Unknown-Test Use
*m*	Top 1/8 of the channels of the canonical grid, i.e., m=15 of $N_{z}=120$ (75 m at ∆z=5 m in the controlled array); the same 1/8 rule is applied to every multichannel corpus after resampling	Prespecified physical footprint	None
$c_{\rho },s_{\rho }$	0.30,0.06	Fixed membership center and width	None
$c_{\kappa },s_{\kappa }$	0.55,0.07	Fixed membership center and width	None
$c_{s},s_{s}$	3.0 dB, 1.5 dB	Fixed SNR membership center and width	None
τ	0.4; the sweep in Section 6.3.5 is a post hoc sensitivity analysis	Fixed gate setting	Not selected from the sweep
$\lambda_{\alpha }$	Augmented order statistic in Equation (6)	Calibrated on verified gate-passing negatives	None in source-static tests
η	90th percentile of the novelty score ν; in the controlled testbed it is taken over gated non-alarming known calibration windows, whereas in the public-corpus runs it is taken over the whole known-class calibration split	Calibrated on known-class calibration data	None
Recognizers	Controlled testbed and sim-to-real: 300-tree RF (min_samples_leaf = 2); public corpora: 400-tree RF (min_samples_leaf = 2); 2-D CNN: 16/32/64 channels, 14 Adam epochs	Known-class training data	None

**Table 2:** Public DAS field corpora and leave-classes-out open-world splits used in Section 6.2.

Corpus	Geometry	Channels	Held-Out Novel Threats	Held-Out Novel Negatives
BJTU ϕ-OTDR [34]	Buried fiber	12/window	Walking	Watering
OptaSense DAS [35]	Perimeter	1700 → 160	Running, construction	Longboard roll, gate open/close
Mendeley DAS [36]	Single channel	1	—	Vehicle

Known classes—BJTU: background, digging, knocking, shaking; OptaSense: vehicle, fence, walking (seven of the eight release categories retained, fibre manipulation unavailable in the processed subset); Mendeley: hammering, background. BJTU “watering” is treated as a benign nuisance rather than an intrusion, so it forms a held-out novel-negative stratum; under the source taxonomy’s broader intrusion grouping it would instead be a held-out threat, in which case BJTU supplies no novel-negative stratum and ${\mathrm{FAR}}_{\mathrm{nov}}$ is undefined for that corpus.

**Table 3:** PCRC on three public DAS field datasets at α=0.05 (Wilson 95% confidence intervals, CIs, where shown). ${\mathrm{FAR}}_{\mathrm{kn}}$/${\mathrm{FAR}}_{\mathrm{nov}}$: known-/novel-negative false-alarm rate; ${\mathrm{Rec}}_{\mathrm{kn}}$/${\mathrm{Cov}}_{\mathrm{nov}}$: known-threat auto-alarm recall/novel-threat three-tier coverage. A2 reject is the fraction of novel negatives rejected as physically inadmissible. The last column is the novel-negative FAR after adaptive recalibration. A dash means the corresponding novel-threat or novel-negative stratum is absent; “n/a” means the spatial gate is unavailable. The ${\mathrm{FAR}}_{\mathrm{nov}}$ and ${\mathrm{FAR}}_{\mathrm{nov}}^{\mathrm{recal}}$ columns are both measured on the disjoint evaluation half of the novel-negative pool used by the recalibration experiment (125 windows on BJTU, 220 on OptaSense, 1244 on Mendeley); the static rates over the full pool are 0.888[0.843,0.921], 0.323[0.281,0.368], and 0.981[0.975,0.985], respectively. Entries marked ^†^ are known-class quantities computed under the window-level split of Section 6.1; they are optimistic on all three corpora because recording identifiers are not preserved (and OptaSense windows overlap by 25%), and must not be read as deployment generalization. The held-out-class columns (${\mathrm{FAR}}_{\mathrm{nov}}$, ${\mathrm{Cov}}_{\mathrm{nov}}$, A2, ${\mathrm{FAR}}_{\mathrm{nov}}^{\mathrm{recal}}$) are unaffected by this mechanism.

Dataset	${\mathrm{FAR}}_{\mathrm{kn}}$	${\mathrm{FAR}}_{\mathrm{nov}}$	${\mathrm{Rec}}_{\mathrm{kn}}$	${\mathrm{Cov}}_{\mathrm{nov}}$	A2	${\mathrm{FAR}}_{\mathrm{nov}}^{\mathrm{recal}}$
BJTU (12 ch)	0.000 ^†^	0.896	0.866 ^†^	0.868	0.000	0.072
OptaSense (160 ch)	0.000 ^†^	0.309	0.798 ^†^	0.675	0.000	0.050
Mendeley (1 ch)	0.037 ^†^	0.983	0.985 ^†^	—	n/a	0.080

**Table 4:** Open-world alarm filtering vs. target FAR α. “Classifier” is the recognizer’s threat posterior with a conformal threshold calibrated on known negatives; PCRC adds the physics gate. FAR is reported separately on known and unknown negatives; recall is reported on known and unknown threats. The zero values for PCRC on unknown negatives are conditional on the simulated unknown nuisance classes satisfying A2.

	Classifier + Conformal				PCRC (Ours)			
α	${\mathrm{FAR}}_{\mathrm{kn}}$	${\mathrm{FAR}}_{\mathrm{unk}}$	${\mathrm{Rec}}_{\mathrm{kn}}$	${\mathrm{Rec}}_{\mathrm{unk}}$	${\mathrm{FAR}}_{\mathrm{kn}}$	${\mathrm{FAR}}_{\mathrm{unk}}$	${\mathrm{Rec}}_{\mathrm{kn}}$	${\mathrm{Rec}}_{\mathrm{unk}}$
0.01	0.009	0.422	1.000	0.530	0.001	0.000	0.998	0.500
0.02	0.013	0.455	1.000	0.540	0.004	0.000	0.998	0.505
0.05	0.053	0.480	1.000	0.650	0.012	0.000	0.998	0.540
0.10	0.108	0.532	1.000	0.748	0.033	0.000	0.998	0.620
0.15	0.172	0.585	1.000	0.873	0.046	0.000	0.998	0.680
0.20	0.215	0.637	1.000	0.943	0.063	0.000	0.998	0.733

**Table 5:** Three-tier (Alarm+Review) coverage of PCRC. Review load is the fraction of *negatives* sent to review (spurious load).

α	Cov. Known Thr.	Cov. Unknown Thr.	Review Load (Neg.)
0.01	0.998	0.695	0.034
0.02	0.998	0.695	0.032
0.05	0.998	0.695	0.027
0.10	0.998	0.695	0.013
0.15	0.998	0.695	0.004
0.20	0.998	0.733	0.000

**Table 6:** Ablation at α=0.05. ${\mathrm{FAR}}_{\mathrm{unk}}$/${\mathrm{FAR}}_{\mathrm{all}}$ on negatives; recall on known/unknown threats.

Variant	${\mathrm{FAR}}_{\mathrm{unk}}$	${\mathrm{FAR}}_{\mathrm{all}}$	${\mathrm{Rec}}_{\mathrm{kn}}$	${\mathrm{Rec}}_{\mathrm{unk}}$
Fixed threshold (0.5)	0.025	0.009	1.000	0.500
Conformal only	0.480	0.197	1.000	0.650
Physics gate + fixed	0.000	0.001	0.998	0.500
PCRC (full)	0.000	0.008	0.998	0.540

**Table 7:** PCRC vs. open-set/OOD rejection on the CNN backbone (α=0.05, mean over three seeds; std ≤0.08 except Energy ${\mathrm{Rec}}_{\mathrm{unk}}$ 0.20). “None” is classifier+conformal without rejection. Last column is known-vs-unknown novelty-detection AUROC.

Accept Rule	${\mathrm{FAR}}_{\mathrm{kn}}$	${\mathrm{FAR}}_{\mathrm{unk}}$	${\mathrm{Rec}}_{\mathrm{kn}}$	${\mathrm{Rec}}_{\mathrm{unk}}$	AUROC
None (no rej.)	0.057	0.824	1.000	0.967	0.500
MSP [13]	0.050	0.132	0.868	0.384	0.880
Energy [15]	0.053	0.220	0.829	0.506	0.819
OpenMax [10]	0.048	0.136	0.874	0.396	0.879
Mahalanobis [16]	0.052	0.322	0.874	0.497	0.728
PCRC (ours)	0.018	0.000	1.000	0.975	0.513

**Table 8:** Sim-to-real transfer (α=0.05, mean over three seeds). Train on source, test on shifted field domain. SRC: source-calibrated $\lambda_{\alpha }$; FLD: $\lambda_{\alpha }$ recalibrated online on verified field negatives.

Method	${\mathrm{FAR}}_{\mathrm{kn}}$	${\mathrm{FAR}}_{\mathrm{unk}}$	${\mathrm{Rec}}_{\mathrm{kn}}$	${\mathrm{Rec}}_{\mathrm{unk}}$
Classifier (SRC)	0.479	0.882	1.000	0.737
Classifier (FLD)	0.049	0.078	0.910	0.433
PCRC (SRC)	0.146	0.000	0.698	0.283
PCRC (FLD)	0.016	0.000	0.660	0.160

**Table 9:** Field-drift stress test (α=0.05, pooled over five seeds, Wilson 95% CIs). A static source-calibrated λ loses FAR validity under drift; online sliding-window recalibration on verified negatives restores target-level FAR on known negatives. The simulated novel negatives remain below the physics threshold throughout.

Quantity	Static λ	Online Recal. λ
Known-neg. FAR	0.111 [0.104,0.118]	0.033 [0.029,0.037]
Novel-neg. FAR	0.000 [0.000,0.001]	0.000 [0.000,0.001]
Strong-threat recall	0.659 [0.644,0.673]	0.585 [0.570,0.600]
Strong-threat missed rate	0.341 [0.327,0.356]	0.415 [0.400,0.430]
Weak-threat recall	0.618 [0.604,0.633]	0.618 [0.604,0.633]
Weak-threat missed rate	0.382 [0.367,0.396]	0.382 [0.367,0.396]
Review load (neg.)	0.043 [0.040,0.047]	0.095 [0.090,0.101]

## Data Availability

The analysis scripts (Python 3.12.2; NumPy 2.4.4; SciPy 1.17.1; Matplotlib 3.10.8; scikit-learn 1.9.0; and PyTorch 2.12.1) and the metrics files (JSON) that reproduce the reported experiments are available from the corresponding author upon reasonable request. The public datasets used in this study are the BJTU buried-fiber ϕ-OTDR event dataset [34] (https://github.com/BJTUSensor/Phi-OTDR_dataset_and_codes, accessed on 8 August 2026), the DAS event-classification dataset [35] (DOI: 10.1038/s41597-025-05088-4), and the Mendeley Data DAS dataset [36] (DOI: 10.17632/fp77d4223z.1).

## Supplementary Materials

The following supporting information can be downloaded at https://www.mdpi.com/article/10.3390/s26165174/s1, Table S1: Per-class mean physical descriptors and physical-consistency scores; Table S2: Two-dimensional CNN backbone results; Table S3: Five-seed robustness results; Table S4: Sensitivity to the physics-gate threshold; Table S5: Confusion matrix on known test classes; Table S6: Forced closed-set predictions for held-out classes; Figure S1: Unknown-negative false-alarm rate for the two-dimensional CNN backbone; Figure S2: Unknown-negative false-alarm rate over five independent seeds; Figure S3: Sensitivity to the physics-gate threshold.

## Abbreviations

The following abbreviations are used in this manuscript:

AUROC	Area under the receiver operating characteristic curve
BJTU	Beijing Jiaotong University
CFAR	Constant false-alarm rate
CI	Confidence interval
CNN	Convolutional neural network
DAS	Distributed acoustic sensing
DOFS	Distributed optical fiber sensing
ECE	Expected calibration error
FAR	False-alarm rate
MSP	Maximum softmax probability
OOD	Out-of-distribution
PDE	Partial differential equation
PCRC	Physically Consistent Risk Calibration
RF	Random forest
SNR	Signal-to-noise ratio
SVM	Support vector machine
TPR	True-positive rate
WPD–EMD	Wavelet packet decomposition–empirical mode decomposition
ϕ-OTDR	Phase-sensitive optical time-domain reflectometry
