# Supplementary material for: Physically Consistent Risk Calibration for Open-World Alarm Filtering in Distributed Optical Fiber Sensing
Source: Sensors (Basel). 2026 Aug 15;26(16):5174. doi: 10.3390/s26165174 (PMC13517819; doi:10.3390/s26165174)
Supplement: Supplementary file 1 [file sensors-26-05174-s001.zip › sensors-4457138-supplementary.pdf]

sensors

MDPI

Article

Supplementary Materials for Physically Consistent Risk Calibration for Open-World Alarm Filtering in Distributed Optical Fiber Sensing

Qingmin Hou [1], Hanyang Zhang [2], Guanghua Xiao [1], Peng Zhang [3] and Ziguang Jia [4,*]

[1] School of Smart City, Chengdu Vocational & Technical College of Industry, Chengdu 610213, China; sspawn@163.com (Q.H.); xiaoguanghua_hit@163.com (G.X.)
[2] College of Computer Science, Sichuan University, Chengdu, Sichuan 610065, China; 2024323042001@stu.scu.edu.cn (H.Z.)
[3] Department of Civil Engineering, Dalian Maritime University, Dalian 116026, China; peng.zhang47@dlmu.edu.cn (P.Z.)
[4] School of Chemical Engineering, Ocean and Life Sciences, Dalian University of Technology, Panjin 124221, China; jiaziguang@dlut.edu.cn (Z.J.)
* Correspondence: jiaziguang@dlut.edu.cn

Keywords: distributed optical fiber sensing; open-world recognition; false-alarm rate; robustness; sensitivity analysis

S1. Descriptor Diagnostics

Table S1 reports the per-class physical descriptors used to audit the compactness, common-mode, and signal-to-noise components of the physical-consistency score. The values are descriptive diagnostics rather than additional fitted parameters.

Table S1. Per-class mean physical descriptors and physical-consistency score for 150 windows per class of the controlled testbed, computed on the canonical 120×200 grid with $m = 15$ dominant channels and the membership settings of Table 1 of the main text. Each entry is the mean over windows of the corresponding per-window quantity; because P is a nonlinear function of the descriptors, the tabulated P is not exactly the value obtained by substituting the tabulated ρ, κ, and SNR into Equation (5) of the main text. The score is high only for admissible near-fiber disturbances.

Class	Threat	Known	ρ	κ	SNR	P
background	0	✓	0.15	0.12	1.0	0.26
wind/rain	0	✓	0.21	0.85	2.4	0.11
vehicle	0	✓	0.84	0.23	17.3	1.00
walking	1	✓	0.27	0.15	4.1	0.63
digging	1	✓	0.72	0.18	12.8	1.00
drilling	1	—	0.86	0.16	16.6	1.00
climbing	1	—	0.27	0.14	3.9	0.61
animal	0	—	0.16	0.12	1.1	0.27
elec. spike	0	—	0.15	0.73	0.8	0.10

S2. Backbone Robustness

A compact two-dimensional convolutional neural network was evaluated to check whether the PCRC behavior depends on the random-forest recognizer used in the primary experiment. Table S2 and Figure S1 show the corresponding unknown-negative false-alarm rate and unknown-threat recall.

 https://doi.org/10.3390/s26165174

Table S2. Two-dimensional CNN backbone results, reported as the mean over three seeds. Known-negative false-alarm rate remains at or below the target and known-threat recall is 1.000 for both methods.

	CNN + Conformal		CNN + PCRC	
α	FAR_{unk}	Rec_{unk}	FAR_{unk}	Rec_{unk}
0.01	0.632	0.930	**0.000**	0.903
0.02	0.699	0.938	**0.000**	0.938
0.05	0.818	0.958	**0.000**	0.967
0.10	0.882	0.967	**0.000**	0.978
0.15	0.937	0.973	**0.000**	0.990
0.20	0.963	0.976	**0.000**	0.993

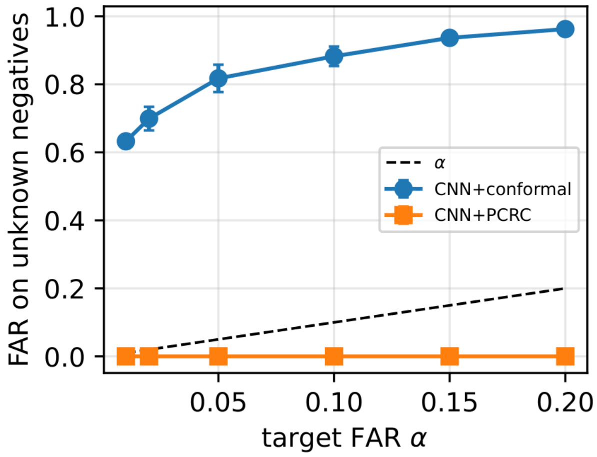

Figure S1. Unknown-negative false-alarm rate for the two-dimensional CNN backbone, shown as mean plus or minus one standard deviation over three seeds. The conformal-on-classifier baseline gives values from 0.63 to 0.96, whereas CNN plus PCRC rejects the simulated inadmissible nuisance classes.

S3. Multi-Seed Robustness

The primary random-forest protocol was repeated over five independent seeds. Table S3 and Figure S2 report the resulting variation in unknown-negative false-alarm rate and threat recall.

Table S3. Five-seed robustness results, reported as mean plus or minus standard deviation.

α	FAR_{unk} (Classifier)	FAR_{unk} (PCRC)	Rec_{kn} (PCRC)	Rec_{unk} (PCRC)
0.01	0.385 ± 0.067	$\mathbf{0.000 \pm 0.000}$	0.992	0.499
0.05	0.475 ± 0.016	$\mathbf{0.000 \pm 0.000}$	0.999	0.562
0.10	0.518 ± 0.019	$\mathbf{0.000 \pm 0.000}$	0.999	0.643
0.20	0.620 ± 0.014	$\mathbf{0.000 \pm 0.000}$	0.999	0.764

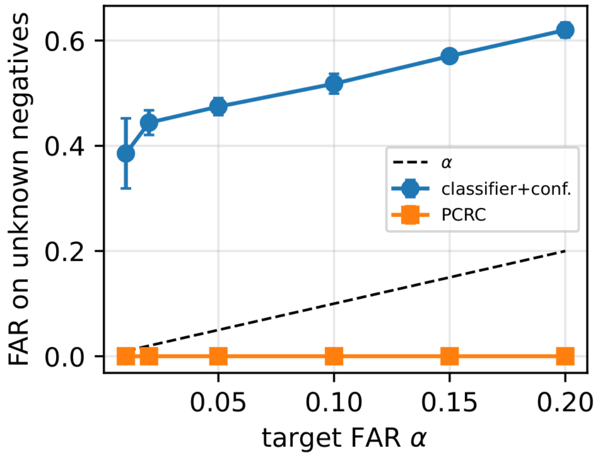

Figure S2. Unknown-negative false-alarm rate over five independent seeds, shown as mean plus or minus one standard deviation for the random-forest backbone.

S4. Physics-Gate Sensitivity

The physics-gate threshold was varied while the target false-alarm rate was held at 0.05. Table S4 and Figure S3 show the trade-off between rejecting inadmissible nuisance windows and retaining weak threats.

Table S4. Sensitivity to the physics-gate threshold at a target false-alarm rate of 0.05, reported as the mean over five seeds.

τ	$\mathbf{FAR_{unk}}$	$\mathbf{Rec_{kn\,thr}}$	$\mathbf{Rec_{unk\,thr}}$
0.10	0.201	1.000	0.641
0.20	0.027	1.000	0.619
0.30	0.002	1.000	0.561
0.40	0.000	0.999	0.562
0.50	0.000	0.958	0.513
0.60	0.000	0.818	0.345
0.70	0.000	0.639	0.148

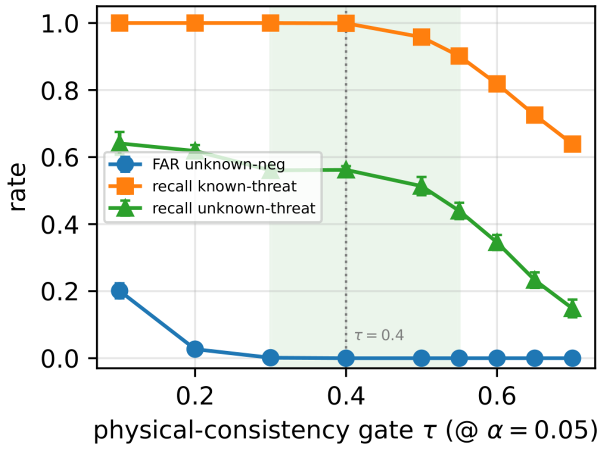

Figure S3. Sensitivity to the physics-gate threshold at a target false-alarm rate of 0.05, shown as mean plus or minus one standard deviation over five seeds. The shaded interval from 0.30 to 0.55 preserves low unknown-negative false-alarm rate while retaining most known-threat recall; the default threshold is 0.40.

https://doi.org/10.3390/s26165174

S5. Class-Wise Confusion on the Controlled Testbed

Tables S5 and S6 report the class-wise behavior of the recognizer on the controlled testbed, obtained from a paired seeded re-run of the archived experiment (global generator seed 2026, random-forest seed 0); the re-run reproduces the archived aggregate metrics (closed-set accuracy 0.999, in-distribution ECE 0.024, mean unknown-class MSP 0.845) exactly, so the tables below correspond to the reported runs. Table S5 confirms that the high closed-set accuracy is uniform across the five known classes (a single vehicle window is confused with digging). Table S6 shows where the same recognizer places the four held-out classes when forced to choose among known classes: each unknown class collapses almost entirely onto one known class, with high confidence for the physically admissible classes (drilling is labeled vehicle on 200 of 200 windows at mean MSP 0.911; climbing is labeled walking on 200 of 200 at 0.976). This is the class-level mechanism behind the open-set failure quantified in Section 6.3.1 of the main text: closed-set confidence carries no information about novelty.

Table S5. Confusion matrix of the recognizer on known test classes of the controlled testbed (rows: true class; columns: predicted class; 260 windows per class).

True class	backgr.	wind/ rain	vehicle	walking	digging	Recall
background	260	0	0	0	0	1.000
wind/rain	0	260	0	0	0	1.000
vehicle	0	0	259	0	1	0.996
walking	0	0	0	260	0	1.000
digging	0	0	0	0	260	1.000

Table S6. Forced closed-set predictions for the four held-out classes (200 windows each): dominant predicted class and mean maximum softmax probability (MSP).

Held-out class	Dominant prediction	Fraction	Mean MSP
drilling (threat)	vehicle	200/200	0.911
climbing (threat)	walking	200/200	0.976
animal	background	199/200	0.967
elec. spike	wind/rain	165/200	0.524

https://doi.org/10.3390/s26165174
